# Expression of calcification‐related ion transporters during blue mussel larval development

**DOI:** 10.1002/ece3.5287

**Published:** 2019-05-29

**Authors:** Kirti Ramesh, Tejaswi Yarra, Melody S. Clark, Uwe John, Frank Melzner

**Affiliations:** ^1^ GEOMAR Helmholtz Centre for Ocean Research Kiel Germany; ^2^ Department of Biological and Environmental Sciences, Sven Lovén Centre for Marine Infrastructure‐Kristineberg University of Gothenburg Fiskebäckskil Sweden; ^3^ British Antarctic Survey Natural Environment Research Council Cambridge UK; ^4^ Ashworth Laboratories, Institute of Evolutionary Biology University of Edinburgh Edinburgh UK; ^5^ Ecological Chemistry Alfred‐Wegener‐Institut Helmholtz‐Zentrum für Polar‐und Meeresforschung Bremerhaven Germany; ^6^ Helmholtz‐Institute for Functional Marine Biodiversity Oldenburg Germany

**Keywords:** biomineralization, bivalves, gene expression, larvae

## Abstract

The physiological processes driving the rapid rates of calcification in larval bivalves are poorly understood. Here, we use a calcification substrate‐limited approach (low dissolved inorganic carbon, *C*_T_) and mRNA sequencing to identify proteins involved in bicarbonate acquisition during shell formation. As a secondary approach, we examined expression of ion transport and shell matrix proteins (SMPs) over the course of larval development and shell formation. We reared four families of *Mytilus edulis* under ambient (ca. 1865 µmol/kg) and low *C*_T_ (ca. 941 µmol/kg) conditions and compared expression patterns at six developmental time points. Larvae reared under low *C*_T_ exhibited a developmental delay, and a small subset of contigs was differentially regulated between ambient and low *C*_T_ conditions. Of particular note was the identification of one contig encoding an anion transporter (SLC26) which was strongly upregulated (2.3–2.9 fold) under low *C*_T_ conditions. By analyzing gene expression profiles over the course of larval development, we are able to isolate sequences encoding ion transport and SMPs to enhance our understanding of cellular pathways underlying larval calcification processes. In particular, we observe the differential expression of contigs encoding SLC4 family members (sodium bicarbonate cotransporters, anion exchangers), calcium‐transporting ATPases, sodium/calcium exchangers, and SMPs such as nacrein, tyrosinase, and transcripts related to chitin production. With a range of candidate genes, this work identifies ion transport pathways in bivalve larvae and by applying comparative genomics to investigate temporal expression patterns, provides a foundation for further studies to functionally characterize the proteins involved in larval calcification.

## 1 INTRODUCTION

During bivalve ontogeny, major developmental steps occur rapidly within the first days of life to produce the larval shell, prodissoconch I (PD I). Successful deposition of the PD I shell is essential for subsequent development as the calcified shell provides structural support for swimming and feeding (Galtsoff, 1964). Calcification begins as the precipitation of a calcium carbonate shell onto an organic template during the trochophore larval stage which is characterized by a free‐swimming, ciliated larva (Bayne, 1976). The supply of calcification substrates (Ca^2+^ and HCO_3_^−^) to the site of calcification by means of transepithelial transport is crucial (Ramesh, Hu, Thomsen, Bleich, & Melzner, 2017). In addition, organic matrix components such as shell matrix proteins and carbohydrates (up to 5% of shell mass, Simkiss & Wilbur, 2012) are synthesized and incorporated into the growing shell, providing the organic template for Ca^2+^ deposition. Although comprising a small proportion of the shell content, shell matrix proteins are a diverse assemblage of proteins that are important for polymorph control, crystal nucleation, deposition kinetics, and structure (Marin, Luquet, Marie, & Medakovic, 2008; Miyamoto et al., 2013). Within 2 days of development, bivalve larvae precipitate an inorganic aragonitic shell that is almost equivalent to their somatic mass and covers the entire larval body (Waldbusser et al., 2013). Accompanying this transition into PD I larval stage is a 250‐fold increase in larval calcium content within a few hours (Ramesh et al., 2017). In bivalve larvae, the substrates for calcification (Ca^2+^ and HCO_3_^−^) are not accumulated prior to calcification but are continually supplied during larval shell formation, most likely by means of transepithelial transport (Ramesh et al., 2017).

The transport of calcium and bicarbonate ions to, and removal of protons from, the calcification space in bivalves is regulated by the expression and activity of membrane‐bound ion transport proteins (Ramesh et al., 2017; Sillanpaa, Sundh, & Sundell, 2018). The close relationship between calcification and acid–base‐related ion transport processes makes it difficult to distinguish between these processes as intracellular pH regulation is also achieved via movement of protons and bicarbonate across the cell membrane. Primary active ion transporters such as V‐type H^+^ ATPases and H^+^/K^+^‐ATPases utilize energy (ATP) for proton translocation. Secondarily active proton transport is driven by the electrochemical gradient provided by other membrane‐bound transport proteins, often the ubiquitous Na^+^/K^+^ ATPase (NKA). These secondarily active proteins are important for pH_i_ regulation and are involved in the translocation of protons and bicarbonate via Na^+^‐coupled pathways (Na^+^/H^+^ exchangers SLC9, Na^+^ HCO_3_^−^ cotransporters, SLC4), anion exchangers (Cl^−^/HCO_3_^−^ exchangers, SLC4), or voltage‐gated proton channels. The cellular pathways elicited to regulate intracellular pH are fundamentally conserved in eukaryotes, and the presence of all relevant transporter families in bivalves has been confirmed by genome and transcriptome sequencing projects in the last decade (Li, Sun, et al., 2016; Murgarella et al., 2016; Takeuchi et al., 2012; Zhang et al., 2012). Several of these membrane‐bound proteins have been functionally characterized to be involved in mollusc intracellular pH regulation (Boron & DeWeer, 1976; Ellington, 1993; Sillanpaa et al., 2018; Zange, Grieshaber, & Hans, 1990). However, the role and regulation of the ion transport proteins that facilitate substrate uptake and proton extrusion have not yet been characterized in mollusc larval calcification. While there are a number of transcriptome sequencing studies available that challenged adult and larval mollusc calcification processes using future ocean acidification scenarios, there are no published accounts of direct functional characterization of putative calcification‐relevant ion transporters using knock down or knock out techniques (DeWit, Durland, Ventura, & Langdon, 2018; Goncalves et al., 2016; Hüning et al., 2013). On the other hand, a number of studies have successfully used RNAi to demonstrate functional roles of specific shell matrix proteins and have shown that their presence is critical to “normal” shell development (Fang et al., 2011; Funabara et al., 2014; Suzuki et al., 2009). Previous studies on mollusc larval development have revealed several shell matrix proteins and genes associated with shell production (Li, Zhang, et al., 2016; Liu et al., 2015) and indicate that bivalve larvae utilize notably different shell matrix proteins when compared with adults (Zhao et al., 2018).

The absence of information on the fundamental mechanisms of bivalve larval calcification physiology limits our ability to predict how these organisms can respond and adapt to environmental change. One way to address hypotheses regarding the role of various ion transporters related to mineral formation and organic deposition in the larval calcification pathway is by challenging calcification in a substrate‐limited environment. In this study, we used RNA‐Seq techniques to identify ion transport and shell matrix proteins involved in the different developmental stages of calcifying larvae of the blue mussel, *Mytilus edulis*. We hypothesized that the genes involved in calcification in *M. edulis* would exhibit severe changes in expression related to the rapid rates of PD I calcification in these organisms. Based on previous empirical data on larval mussels which demonstrated that limiting conditions of dissolved inorganic carbon (*C*_T_) elicited strong reductions in larval calcification (Thomsen, Haynert, Wegner, & Melzner, 2015), we used a substrate‐limited approach (low *C*_T_) to gain insight into the role of these genes, primarily those involved in inorganic carbon acquisition and crystal formation.

## 2 MATERIALS AND METHODS

Adult mussels were collected in Kiel Fjord (54°19.8′N; 10°9.0′E) from subtidal depths (ca. 1.5 m) in June 2016. Kiel mytilids are *Mytilus edulis* × *trossulus* hybrids with high *edulis*‐like allele frequencies (Stuckas, Stoof, Quesada, & Tiedemann, 2009). We will refer to them as Baltic *Mytilus edulis*‐like according to Stuckas et al. (2017). Spawning was induced by exposing the adults to rapidly elevated water temperature between 18 and 25°C. Spawning individuals were separated, and gametes were collected in individual beakers filled with 0.2 µm filtered seawater (FSW). Eggs were fertilized with sperm, and fertilization success was estimated by the presence of polar bodies/cell cleavage. Cleaving embryos were reared in 10‐L Duran glass bottles at a density of 10 embryos ml^−1^ bubbled with pressurized air through plastic tubing. All experiments were performed at 17°C. A total of four separate fertilizations were obtained to conduct replicate experiments (*n* = 4).

### 2.1 Experimental treatments and sample collection

Seawater carbonate chemistry was manipulated by the addition of 1 M HCl to FSW, thereby lowering the availability of calcification substrates (HCO_3_^−^, but also CO_3_^2−^). Excess carbon dioxide was removed by aeration with pressurized air for 1 hr. Seawater pH was determined on the NBS scale using a WTW 3310 pH meter equipped with a Sentix 81 electrode. Water for carbonate chemistry samples was collected from the culture bottles just before adding embryos to the bottles. Samples were collected in 52‐mL Duran Schott glass bottles with glass stoppers and preserved by the addition of 10 μl of saturated HgCl_2_ solution. Seawater *C*_T_ was measured using an AIRICA *C*_T_ analyzer (Marianda, Germany) and verified with certified reference material (batch 142; Scripps Institution of Oceanography, University of California, San Diego, CA, USA). Seawater carbonate chemistry parameters were calculated using the CO2SYS program with KHSO_4_, K1, and K2 dissociation constants after Dickson (1990) and Roy et al. (1993), respectively. Cleaving embryos were added to treated water once pH had increased to stable values (ca. 8.14). Temperature, salinity, and carbonate chemistry parameters of experimental conditions are shown in Table S1.

For each experiment, embryos from single fertilizations were added to both control (FSW) and treatment (low *C*_T_) bottles and sampled at six developmental time points as determined by hours postfertilization (hpf). Samples were collected at 20, 22, 24, 27, 30, and 35 hpf from control bottles, based on time points that were previously identified to be critical for calcification (Ramesh et al., 2017). To correct for developmental delay in treatment bottles, samples were collected at similar developmental stages to those in control bottles, as determined by frequent microscopic observation of percentage shell cover of the larval body (Figure S1). Stage 1 occurred prior to calcification while at Stage 2, the onset of calcification was observed, and larvae exhibited a dorsal flattening at the region of the shell field. At Stages 3 and 4, the trochophore shell was observed by the presence of a shell that covered ca. 10% and 49% of the larval body, respectively. Finally, at Stages 5 and 6, larvae had secreted a shell that covered ca. 76% and 100% of the larval body, respectively.

Samples were quickly concentrated on a mesh (55 µm), transferred to 1.5‐ml Eppendorf tubes and centrifuged at 10,000 *g* to form a larval pellet (~6,000 larvae). Seawater was removed using a pipette, and samples were flash frozen in liquid nitrogen and stored at −80°C.

For each sample time point, ca. 100 larvae for photographs were fixed in 4% paraformaldehyde prepared in FSW, buffered to pH 8.2 using 5 mM NaOH. Samples were photographed using a Zeiss Axio Scope A1 microscope equipped with a ProgRes CF Jenoptik camera and ProgRes Capture Pro software (v. 2.9.0.1).

### 2.2 RNA extractions and sequencing

Total RNA was extracted from samples using a RNeasy Mini Kit according to manufacturer's instructions (Catalog no. 74104, Qiagen). RNA yield and purity were initially assessed by measuring A260/A230 and A260/A280 ratio, with a NanoDrop spectrophotometer (NanoDrop2000; Thermo Scientific), followed by integrity analysis on a bioanalyzer (Experion, Bio‐Rad). The libraries were prepared from 1 µg RNA per sample with the TruSeq stranded mRNA HT sample preparation kit (Illumina). The quality and concentration of the resulting libraries were checked with a bioanalyzer (Agilent 2100) using an Agilent DNA 7500 Kit (Agilent Technologies). Library preparation and bioanalyzer validation were performed according to manufacturer protocols. DNA fragment length and concentration data were then used to calculate the molarity of individual libraries, which were subsequently pooled equimolarly (10 nM) and sequenced on an Illumina NextSeq500 sequencer to generate 75 bp single end reads. Illumina BCL files were converted to fastq files and de‐multiplexed using bcl2fastq (v2.17; Illumina) using default settings.

### 2.3 Bioinformatics analysis

All bioinformatics analyses were carried out using default parameters, unless otherwise specified. Illumina adapter trimming of the reads was performed using Trimmomatic v.0.33 (Bolger, Lohse, & Usadel, 2014), and the reads were further trimmed based on quality and length using Fastq‐mcf v.1.04.636 (Aronesty, 2011), setting the Phred quality score to 30 and minimum read length to 60 bp. A published mantle transcriptome of Baltic *M. edulis*‐like individuals (Yarra, 2018, PRJNA494236), collected from the same geographic coordinates as the animals in this study, was used for mapping reads. The cleaned reads were aligned to the Baltic *M. edulis*‐like mantle transcriptome (Yarra, 2018) using Bowtie v.1.1.1 (Langmead, Trapnell, Pop, & Salzberg, 2009), and the digital measure of transcript abundance was calculated using RSEM (RNA‐Seq by Expectation‐Maximization) v.1.2.20 (Li & Dewey, 2011). All contigs with digital expression levels less than 2 counts per million (CPM) at the Trinity “gene” level, in at least half the libraries, were filtered out before analysis for differential expression. Preliminary analysis of the data revealed mislabeling of four samples, and the mislabeling was corrected as discussed in Appendix S1.

Contigs from the mantle transcriptome were annotated with a few different databases (Yarra, 2018). Sequence similarity searches of the transcript sequences were performed using BLAST (Altschul, Gish, Miller, Myers, & Lipman, 1990, blastx) with an E‐value cut off of 1e^−10^ against public databases SwissProt (accessed 08 January, 2017), Trembl (accessed 04 August, 2016), Shell Matrix Protein database (01 JULY 2018; Yarra, 2019, https://doi.org/10/cz2w), and the in‐house transmembrane transporters list.. Matches were considered where at least 40% of the query sequence was aligned to, were considered to reflect strong sequence similarity. Transdecoder (part of the Trinity pipeline) was used to translate contigs into putative protein sequences of at least 20 codons. Translated protein sequences were mined for domain and family information using Interproscan (Jones et al., 2014), and Gene Ontology (GO) terms for contigs were assigned based on the Interpro database (Finn et al., 2017).

Differentially expressed contigs between developmental stages and treatments were identified using edgeR 3.20 (Robinson, McCarthy, & Smyth, 2010). Differential gene expression between the different libraries was assessed using the paired experimental model (Family + Treatment and Stage), and only results with FDR values of at least 0.001 were considered. EBSeqHMM (Leng et al., 2015) was used to assess the expression profile of genes over the developmental stages and to cluster genes by expression paths. The expression profiles of both control and treatment libraries through the development stages were analyzed and compared. Only results with a FDR value of at least 0.001 were considered. For further GO enrichment analysis, only expression profiles with at least a 50% posterior probability (Max PP) were used. Enrichment of GO terms for genes clustered into the same expression profile using EBSeqHMM was performed using downstream Trinity pipeline for Trinotate and GOSeq (Grabherr et al., 2011), and only results with at least FDR value of 0.05 were considered. For the purpose of further characterizing contigs of interest, translated protein sequences were globally aligned to sequences from the public databases using Mafft (Katoh, Rozewicki, & Yamada, 2017), with the BLOSUM62 (Henikoff & Henikoff, 1992) scoring matrix, and neighbor joining (NJ) trees were constructed using the WAG matrix (Whelan & Goldman, 2001) with a bootstrap value of 100, on only the conserved residues between all sequences. The expression profiles for contigs of interest with the highest posterior probability were displayed, along with the normalized count values of all four larval families in the control libraries (with a trend line represented using lowess smoothing). The bicarbonate transport phylogenetic tree was constructed based on 95 conserved sites of 43 sequences, including 10 Baltic *M. edulis*‐like contigs, and a cystic fibrosis transmembrane conductance regulator from zebrafish as an outlier. All accession ID's for protein sequences used in the tree are provided in Table S2.

## 3 RESULTS

### 3.1 Larval development

Manipulation of seawater carbonate chemistry by the addition of 1 M HCl resulted in a reduction of *C*_T_ from 1865.5 ± 26.2 µmol/kg seawater under control conditions to 941.7 ± 51.3 µmol/kg seawater. In addition, a reduction in bicarbonate availability from 1840.8 ± 23.2 to 888.3 ± 47.5 and carbonate availability from 108 ± 3.7 to 43.7 ± 5 was observed (Table S1). Further, seawater *C*_T_ reductions were associated with a decrease in *p*CO_2_ from 423.4 ± 7.2 µatm under control conditions to 244.6 ± 23.7 µatm and Ω_aragonite_ from 1.7 ± 0.03 to 0.6 ± 0.08. Development at reduced *C*_T_ resulted in a developmental delay starting at 22 hpf, corresponding to the onset of calcification (Ramesh et al., 2017, Figure S1, Table 1). The mean developmental delay was 1.71 ± 1.38 hr, and in one family, the delay was observed to go up to 6 hr (Table 1). The variability in developmental delay across replicates may be attributed to biological variability in larval energy budgets or differences in the degree of *M. edulis* × *M trossulus* hybridization between the four replicate families. Morphologically distinct developmental stages were ascribed to Stages 1–6 for further analyses.

**Table 1:** Morphological stages at which Baltic *Mytilus edulis*‐like larvae were sampled during the experiment

Stage	Description	Hours postfertilization				
		Control families	Reared under low *C*_T_ conditions			
			F001	F002	F003	F004
Early trochophore	Precalcification	20	20	20	20	20
Trochophore	Larvae exhibit dorsal flattening at the region of the shell field which marks the onset of calcification	22	22	22	22	22
Trochophore	First trace of mineralization is observed by the presence of a small (ca. 20 µm) shell and birefringence at the hinge area. Presence of an early trochophore shell has previously been observed at 22 hpf (Ramesh et al., 2017)	24	25	25	24	25
Trochophore	49% ± 7.7% of the larval shell is covered by a mineralized shell	27	29	29	28	28
Late trochophore	76.9% ± 7.9% of the larval shell is covered by a mineralized shell	30	33	32	31	31
D‐veliger	Larvae have secreted the PD I shell and exhibit a distinctive “D” shape	35	41	35	36	36

Note

Changes in shell cover are quantified from *N* = 20 larvae and are reported as mean ± *SD*. Under control conditions, larval development across families was relatively uniform (maximum standard deviation = ca. +7% shell cover, Table 1) and sampled at six identical time points.

### 3.2 Quality control of sequencing reads

Sequencing of 48 larval libraries yielded a total of 590 million reads, with 541 million reads remaining after filtering based on quality and length. Cleaned reads were aligned to the Baltic *M. edulis*‐like mantle transcriptome (Yarra, 2018), and mapping rates of approximately 80% were observed for all larval libraries. Filtering based on CPM values yielded 29,177 Trinity genes for further analysis.

### 3.3 Gene expression analysis

Three types of differential gene expression analysis were conducted for this dataset: pairwise comparisons between treatment and control groups at each developmental stage, pairwise comparisons between developmental stages, and time course comparisons between the developmental stages using an auto‐regressive hidden Markov statistical model.

In the pairwise comparisons between treatment and control libraries, very few contigs (53) were found to be differentially expressed at each developmental stage (Table S3).

However, multiple contigs were found to be differentially expressed between the pairwise comparison of developmental stages. A large number of contigs (22,564) were differentially expressed between Stage 1 and each subsequent developmental stage, and multiple contigs were also found to be differentially expressed between each consecutive developmental stage (Table S4). Two hundred and forty‐five contigs were differentially expressed following the onset of larval shell deposition (Stage 2) in comparison to the precalcifying ontogenetic stage (Stage 1; at 20 hpf, Table S5).

The time series‐based differential gene expression analysis revealed two expression paths to be the most prevalent during PD I development in the Baltic *M. edulis*‐like trochophore stage (Table 2). Enrichment of GO terms in the most prevalent expression profile “Down‐Up‐Up‐Up‐Up” (for families reared under control conditions) revealed multiple functions associated with biomineralization, such as calcium ion binding, chitin binding, transmembrane transporter activity, etc. However, very few GO terms were enriched in the second most prevalent expression profile “Up‐Down‐Down‐Down‐Down” (Table 3). Although the absolute number of differentially expressed contigs within the control and treatment libraries was different between developmental stages (Table S4), the enriched GO functionalities were very similar between treatments.

**Table 2:** Top ten expression profiles and number of contigs within each expression profile

Control libraries		Treated libraries	
Expression profile	Num. contigs	Expression profile	Num. contigs
Down‐Up‐Up‐Up‐Up	1,249	Down‐Up‐Up‐Up‐Up	1,242
Up‐Down‐Down‐Down‐Down	837	Up‐Down‐Down‐Down‐Down	867
Up‐Down‐Down‐Down‐Up	492	Down‐Down‐Down‐Down‐Down	447
Down‐Down‐Down‐Down‐Down	470	Down‐Up‐Down‐Down‐Down	393
Down‐Up‐Down‐Down‐Down	357	Down‐Up‐Down‐Down‐Up	354
Down‐Up‐Up‐Up‐Down	333	Down‐Up‐Down‐Up‐Up	294
Down‐Up‐Up‐Down‐Down	137	Down‐Up‐Up‐Up‐Down	291
Up‐Down‐Down‐Up‐Up	105	Up‐Down‐Down‐Down‐Up	205
Down‐Up‐Down‐Down‐Up	99	Up‐Down‐Up‐Down‐Down	191
Up‐Down‐Up‐Up‐Up	92	Up‐Down‐Up‐Up‐Down	173

Note

Only contig expression profiles with at least 0.001 FDR and posterior probability of at least 50% are summarized.

**Table 3:** Enrichment of GO terms in the top two expression profiles

Control libraries	Treated libraries
Profile 1: “Down‐Up‐Up‐Up‐Up”	
Calcium ion binding	Calcium ion binding
Hydrolase activity (O‐glycosyl compounds)	Hydrolase activity (O‐glycosyl compounds)
Transporter activity	Transporter activity
Chitin binding	Chitin binding
Catalytic activity	Catalytic activity
Oxidoreductase activity	Oxidoreductase activity
Polysaccharide binding	Polysaccharide binding
Heme binding	Heme binding
Ion channel activity	Carbohydrate binding
Transmembrane transporter activity	ß‐N‐acetylhexosaminidase activity
Profile 2: “Up‐Down‐Down‐Down‐Down”	
None	Nucleic acid binding
	Microtubule binding
	ATP binding

Note

Top ten Molecular Function (MF) terms with at least 0.05 FDR. Only contig expression profiles with at least 0.001 FDR and posterior probability of at least 50% were used for GOSeq analysis.

### 3.4 Identification of transport pathways involved in calcification

**Figure 1:**
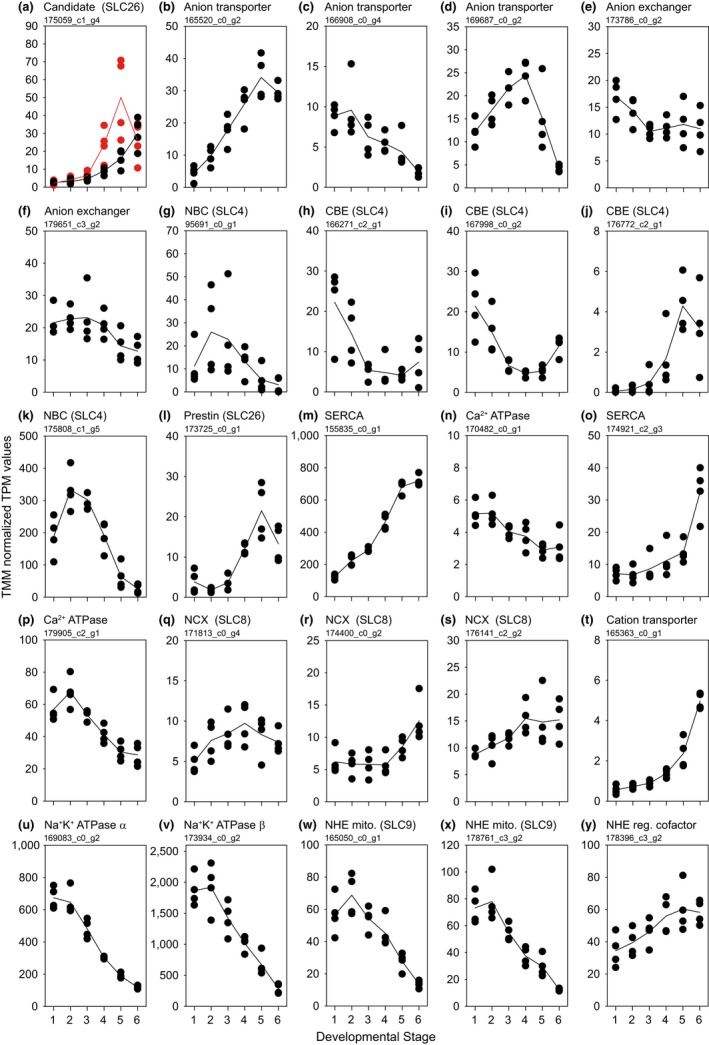
Genes encoding putative ion transport proteins involved in larval calcification in *Mytilus edulis*. The morphological stages described in Table 1 and Figure S1 are marked as 1–6 on the *x*‐axes. Expression values for (a) The candidate SLC26 protein identified by the substrate limitation experiment. Expression values for low *C*_T_ and ambient conditions marked in red in black, respectively. (b–d) Anion transport proteins (e, f) Anion exchange proteins (g, k) A SLC4 sodium bicarbonate cotransporter (h–j) SLC4 chloride‐bicarbonate exchange proteins (l) A SLC26 prestin protein (m, o) Sarco/endoplasmic reticulum calcium ATPase (SERCA) proteins (n, p) Calcium‐transporting ATPase proteins (q–s) Sodium‐calcium exchange proteins (t) Cation‐transporting protein (u) Sodium potassium ATPase α subunit (v) Sodium potassium ATPase β subunit (w, x) Mitochondrial isoforms of sodium‐hydrogen exchange (NHE) proteins from SLC9 and (y) Sodium‐hydrogen exchange (NHE) regulatory cofactor. Maximum posterior probability for all contigs is reported in Table S7

The primary objective of this study was to identify candidates of ion transporter proteins potentially involved in providing substrates (Ca^2+^, HCO_3_^−^) for larval calcification. Substrate (*C*_T_) limitation induced a small set of contigs to be differentially regulated (Table 4, Table S3) with fold change values between 0.26 and 0.57 and between 1.54 and 16.11 for down and upregulated contigs, respectively (Figure S2). Only one contig showed high sequence similarity to an ion transporter (SLC26A11, TRINITY_DN175059_c1_g4) and belongs to solute carrier family 26 (SLC26), a group of ion transport proteins that transport a diverse set of anions, including HCO_3_^−^ (Cordat & Casey, 2009). During the course of Baltic *M. edulis* larval development, expression of this SLC26A11 contig was progressively upregulated under control conditions (Figure 1a, Down‐Up‐Up‐Up‐Up‐‐0.7301 [posterior probability]). The expression of this contig was observed to be 2.3 and 2.9‐fold higher under substrate limitation at Stage 4 and 5, respectively (Table S3).

**Table 4:** Number of differentially expressed contigs in treatment libraries compared to control libraries, at each stage

Stage	Upregulated	Sequence similarity to transmembrane transporters of interest	Downregulated
1	0		0
2	0		0
3	16		0
4	13	Sodium‐independent sulfate anion transporter	1
5	22	Sodium‐independent sulfate anion transporter	1
6	21		5

### 3.5 Putative homology of bicarbonate ion transporters

**Figure 2:**
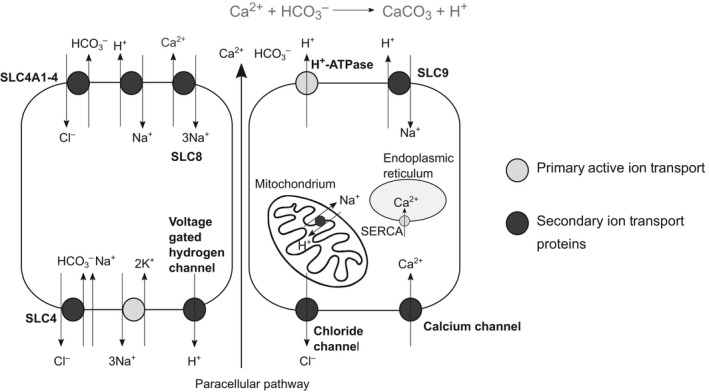
An ion transport model depicting the cellular transport processes of membrane‐bound ion transport proteins exhibiting elevated expression during *Mytilus edulis*‐like larval development. The Na^+^/K^+^ ATPase (NKA) provides the electrochemical gradient for secondary ion transport via proteins such as the Na^+^/H^+^ exchanger (NHE, SLC9), sodium bicarbonate cotransporters (NBC, SLC4), and Na^+^/Ca^2+^ exchangers (NCX, SLC8). Additionally, septate junctions may regulate the permeability of ions via the paracellular pathway (Jonusaite, Kelly, Donini, 2017). The putative precipitation of calcium carbonate in an extracellular calcification space is described in gray. The precise cellular location (apical/basolateral) and distribution of these ion transport proteins are unknown. In situ hybridization studies are necessary to ascertain the expression of these transporters calcifying epithelia

**Figure 3:**
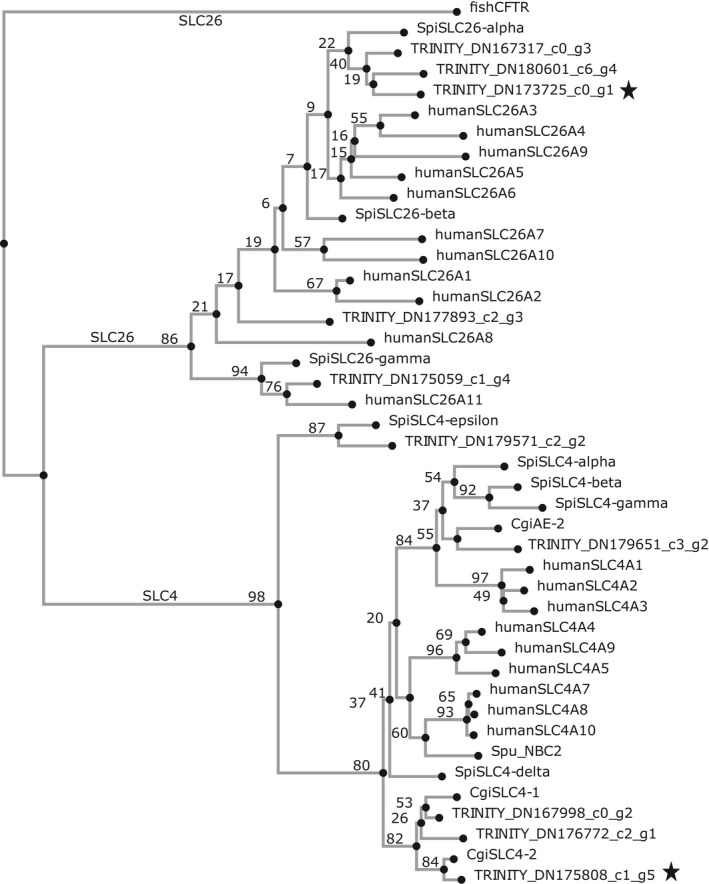
Phylogenetic tree depicting relationships between bicarbonate transporter families in *Homo sapiens* (human), *Stylophora pistillata* (Spi), *Crassostrea gigas* (Cgi), *Strongylocentrotus purpuratus* (Spu), and larval mussels (TRINITY). All sequences, along with accession IDs, are provided in Table S2. Starred sequences were differentially expressed in adult mussels during shell regeneration (Yarra, 2018), and values above the nodes represent bootstrap values

There are multiple families of bicarbonate transporters present in eukaryotes (Alper & Sharma, 2013; Pushkin & Kurtz, 2006). To further characterize the sequence similarity of differentially expressed contigs with bicarbonate‐transporting domains, a phylogenetic tree from multiple sequence alignments of translated protein sequences was assessed (Figure 2). The phylogenetic analysis presented here indicates that like most eukaryotes, Baltic *M. edulis*‐like larvae possess several cellular HCO_3_^−^‐transporting proteins. Two putative mussel HCO_3_^−^‐transporting proteins, which share high sequence similarity with membrane‐bound SLC4 and SLC26 proteins, are the most interesting in the context of the present study (Figure 3). These sequences have been associated with calcification processes as they were found to be upregulated during adult mussel shell repair (Yarra, 2018).

In contrast to the small number of contigs exhibiting differential expression in response to substrate limitation, several contigs putatively encoding ion transport proteins corresponding to solute carrier families SLC4, SLC9, and SLC26 were differentially expressed during the course of larval development and shell deposition (Figure 1). Among these SLC families, several contigs exhibited progressive increases in expression during the course of development (Table S3). These sequences encoded proteins such as sarco/endoplasmic reticulum Ca^2+^‐ATPase, sodium/calcium exchangers (NCX), and the sodium/potassium ATPase. The putative ion transport pathways involved in larval calcification based on expression patterns for contigs of interest are presented schematically in Figure 3.

### 3.6 Shell matrix proteins

Multiple genes that encode shell matrix proteins previously identified in the shell matrices of adult *Mytilus* spp. and expressed by the adult mantle tissue, particularly during shell repair, were found to be differentially expressed during larval shell development. Approximately, 33% of the contigs annotated with SMP domains displayed an increasing expression profile starting from the trochophore stage (Table S6). A few shell matrix proteins (α‐carbonic anhydrase, β‐lactamase, concanavalin A, and cyclophilin PPIase) displayed decreasing expression levels as the initial shell was completed.

## 4 DISCUSSION

In this study, we employed a two‐stage analysis. First, we used a calcification substrate‐limited approach (low dissolved inorganic carbon, *C*_T_) to identify acid–base regulatory proteins necessary for larval calcification. Second, we analyzed gene expression profiles over the developmental time course of *M. edulis* larvae and observed the dynamic expression of several contigs encoding ion transport and shell matrix proteins associated with particular developmental stages. The putative roles of these candidate contigs in acid–base homeostasis and larval calcification are discussed below.

### 4.1 Substrate limitation approach

We used low dissolved inorganic carbon, *C*_T_ to challenge larval acid–base regulatory systems and identify candidate contigs putatively involved in calcification. In comparison to controls, substrate limitation treatments (mean *C*_T_ = 941.7 µmol/kg seawater) were characterized by a ca. 51% and ca. 59% decrease in bicarbonate and carbonate availability, respectively. Such modifications in seawater carbonate chemistry were accompanied by a developmental delay in shell accretion that was consistent with previous empirical data (Thomsen et al., 2015). These observations in developmental delay demonstrated that low *C*_T_ conditions were correlated with decreases in larval calcification rates in *Mytilus edulis* (Thomsen et al., 2015). However, the relatively short developmental delay 1.71 ± 1.38 hr also indicated that larvae are capable of compensating for dramatic reductions in *C*_T_ availability. Developmental delays in response to adverse changes in seawater carbonate chemistry have been reported for several bivalve species (e.g., Ross, Parker, O'Connor, & Bailey, 2011, Timmins‐Schiffman, O'Donnell, Friedman, & Roberts, 2012). However, previous transcriptomic studies have not accounted for such developmental delays (Kelly, Padilla‐Gamino, & Hofmann, 2016). Our results demonstrate the importance of correcting sample collection for developmental delays since we observed that differences in shell morphology (percentage shell cover) were related to specific gene expression profiles, as has been previously demonstrated for larval echinoderms (Stumpp, Dupont, Thorndyke, & Melzner, 2011; Stumpp, Wren, Melzner, Thorndyke, & Dupont, 2011). We identified only 53 contigs to be differentially expressed between control and low *C*_T _conditions. However, median fold change of contigs significantly regulated in low *C*_T_ libraries with respect to control libraries was ca. 2.44 (Up) and 0.52 (Down; Figure S2), indicating that more subtle changes in gene expression were not detected with the present experimental design.

Of particular interest was the 2.32‐ and 2.96‐fold upregulation of one gene (contig TRINITY_DN175059_c1_g4) at Stages 4 and 5 encoding an anion transporter with strong sequence similarity to the solute carrier family 26 (SLC26) members. Solute carrier family 26 members transport a broad group of anions, including HCO_3_^−^, the substrate for larval calcification. Stages 4 and 5 are particularly interesting, as they are characterized by exponential increases in shell deposition, corresponding to a shell cover increase from ca. 10% to ca. 76% of the larval body.

Phylogenetic tree analyses revealed similarity between the *M. edulis* SLC26 contig and the human SLC26A11 sulfate/anion transporter (Figure 3). Recently, the function of SLC26A11 transporters as sodium‐independent sulfate transporters has been critically reviewed based on observations of their function as a chloride channel in mice neurons using electrophysiological techniques (Rahmati et al., 2013). Furthermore, transfection of the SLC26A11 transporter in mouse neuronal cells hints toward the activation of V‐type H^+^‐ATPases by SLC26A11 transporters inducing proton translocation (Rahmati et al., 2013). Alternatively, the upregulation of this transporter during larval development may be related to the uptake of sulfate for synthesis of sulfated macromolecules such as proteoglycans that are present in the organic matrix of mollusc shells (LeRoy & Marie, 2012). Sulfated constituents of the organic matrix in calcified structures have been proposed to play a role in crystal nucleation (Cuif & Dauphin, 2005; Cuif, Dauphin, Doucet, Salome, & Susini, 2003). However, further characterization (heterologous expression) is necessary to verify substrate specificity.

In addition to the SLC26 candidate, the substrate limitation approach also yielded several contigs that encoded proteins with potential roles in *M. edulis* larval calcification, including at least eight differentially regulated contigs with high sequence similarity to transcripts that have been previously demonstrated to be involved in bone mineralization and resorption or have been shown to form components of organic matrices in mammals. For example, mice deficient of transcription factor Sox 8 (upregulated under low *C*_T_ at Stages 5 and 6) exhibit reduced bone mass and impaired osteoblast differentiation (Schmidt et al., 2005). Interestingly, the function of transcription factor Sox 8 is strongly linked to the expression of runt‐related transcription factor 2 (Runx2; Schmidt et al., 2005), where Runx2 (upregulated under low *C*_T_ at Stages 3 and 5) is an important regulator of mammalian bone formation (Fowlkes et al., 2008; Franceschi & Xiao, 2002; Takarada et al., 2013) and arterial calcification (Ruffenach et al., 2016). Two other transcripts putatively encoding tumor necrosis factor α‐inducing proteins and interleukin1 receptor‐associated kinase 4 (IRAK4) that were observed to be upregulated under low *C*_T_ conditions in *M. edulis* larvae have also been associated with osteoblast differentiation and bone resorption (Katsuyama et al., 2014; Tintut, Patel, Parhami, & Demer, 2000). Tumor necrosis factor α is involved in the activation of nuclear factor‐kappa B (NF‐κB, upregulated under low *C*_T_ at Stage 5), a protein whose activation is linked to arterial calcification in humans (Zhao et al., 2012). Substrate limitation also induced upregulation of a contig with sequence similarity to a zinc transporter during Stage 5 of *M. edulis* larval development. In mammals, the knockout of zinc transporters has been demonstrated to result in skeletal disorders (Fukada et al., 2008) and the role of zinc is established in bone mineralization (Yamaguchi, 1998) and mollusc shell deposition (Tan & Mai, 2001). Finally, the expression of a contig encoding putative C‐type lectins was also upregulated under low *C*_T_ in *M. edulis* larvae. C‐type lectins form important components of the shell organic matrix in molluscs (Mann, Edsinger‐Gonzales, & Mann, 2012). For example, the shell matrix protein perlucin that is expressed in *M. edulis* larvae during PD I formation contains a C‐type lectin domain. Therefore, our substrate (*C*_T_) limitation technique elicited an expression change in several contigs that can be related to *M. edulis* larval development.

The low number of differentially expressed contigs with respect to substrate limitation was certainly linked to the high variability in gene expression, as observed before (Hüning et al., 2013; Yarra, 2018), but was also in line with previous studies on calcifying larvae that observe no significant changes in gene expression in response to simulated ocean acidification (Evans, Chan, Menge, & Hofmann, 2013; Kelly et al., 2016). In contrast, adult bivalves have been observed to exhibit differential regulation of genes related to ion and acid–base regulation in response to elevated seawater *p*CO_2_ (Li, Huang, et al., 2016). Differences in larval and adult transcriptomic responses to CO_2_ in other studies may reflect differences in acclimation *p*CO_2_, experimental design and individual variability. Alternatively, acid–base regulation may be ensued via posttranslational mechanisms, for example, the translocation of membrane‐bound transport proteins to compensate for increased transport of calcification substrates (Roa, Munévar, & Tresguerres, 2014; Tresguerres, Parks, Wood, & Goss, 2007) and phosphorylation of ion transport proteins (Flemmer et al., 2010; Levitan, 1994) that can induce their rapid activation (Ramnanan & Storey, 2006; Rapoport & Murad, 1983). Additionally, mapping of data to a larval transcriptome may enable a deeper insight of differentially expressed transcripts that are nonmantle specific, whereas the present study utilized a transcriptome assembled for the adult Baltic *M. edulis*‐like mantle tissue.

### 4.2 Developmental time course analyses

#### 4.2.1 HCO_3_^−^ transport

In eukaryotes, the transport of HCO_3_^−^ may occur via two possible families of membrane‐bound transport proteins, the SLC4 and SLC26 transporters (Alper & Sharma, 2013; Pushkin & Kurtz, 2006). Within the group of SLC4 transporters, proteins are characterized into three major groups, based on mechanism of action: Cl^−^/HCO_3_^−^ exchangers (also known as anion exchangers (AE), Na^+^ − HCO_3_^−^ cotransporters (NBCs) and Na^+^‐driven Cl^−^/HCO_3_^−^ exchangers (NDCBE; Romero, Chen, Parker, & Boron, 2013). The Cl^−^/HCO_3_^−^ exchangers are electroneutral and exchange Cl^−^ and HCO_3_^−^ at 1:1 stoichometry, while the NBCs may function at a Na^+^:HCO_3_^−^ stoichiometry of 1:3/1:2 (electrogenic) or 1:1 (electroneutral; Romero et al., 2013). The SLC26 family of transport proteins (as discussed above) transports a variety of anions including HCO_3_^−^, sulfate (SO_4_^2−^), oxalate, and others and may similarly also be functionally characterized into various groups depending on stoichiometry (Soleimani, 2013). Therefore, depending on which HCO_3_^−^‐transporting protein is utilized, Cl^−^ or Na^+^ is required to provide the electrochemical gradient required for HCO_3_^−^ transport. The provision of such gradients through Na^+^/K^+^‐ATPase is discussed in the following sections. However, if Cl^−^ was the coupled ion for HCO_3_^−^ acquisition during larval calcification, Cl^−^ gradients may be maintained via proton exchange, cation‐coupled Cl^−^ exchange (Na^+^‐K^+^‐2Cl^−^ cotransporters, SLC12) and Cl^−^ channels.

Among all the contigs putatively encoding ion transport and shell matrix proteins investigated in this study, a sequence encoding an NBC exhibited high transcript abundance during the larval development of *M. edulis* with a peak in expression during early calcification (Figure 1k, TRINITY_DN175808_c1_g5). The peak in expression of NBC encoding contigs is accompanied by the onset of shell formation. Following early trochophore development, expression levels of this NBC sequence rapidly decreased. Within the contigs encoding HCO_3_^−^ transport in the transcriptome that exhibited differential expression during larval development, two contigs clustering with SLC4 (TRINITY_DN167998_c0_g2) and SLC26 (TRINITY_DN173725_c0_g1) families were also observed to be upregulated during induced shell repair in adult *Mytilus edulis*, further supporting the role of these transcripts in substrate acquisition for calcification (Yarra, 2018).

#### 4.2.2 Ca^2+^ transport

Prior to the onset of calcification at the trochophore larval stage, Ca^2+^ is not accumulated and stored by mussel larvae (Ramesh et al., 2017). Rapid calcification of the PD I shell in mussels is accompanied by a tremendous uptake of calcium by larvae within a few hours (Ramesh et al., 2017). In contrast to larval sea urchins (Vidavsky et al., 2016; Vidavsky, Masic, Schertel, Weiner, & Addadi, 2015)the acquisition of the calcification substrates Ca^2+^ and HCO_3_^−^ from seawater via endocytotic transport does not seem to be a major pathway for calcium acquisition in larval mussels, suggesting that uptake of Ca^2+^ likely occurs via transepithelial pathways (Ramesh et al., 2017). Our study indicated that in *M. edulis*, four transcripts (sarco/endoplasmic reticulum Ca^2+^‐ATPase [SERCA], Ca^2+^‐ATPases, Ca^2+^ channels, and sodium/calcium exchangers [NCX]) were involved in Ca^2+^ transport during larval development (Figure 1m–s). Expression of contigs encoding these four Ca^2+^ transport proteins was upregulated as ontogenetic development progressed, with largest increases in expression for contigs encoding SERCA and NCX, suggesting a pivotal role of these transporters. In mammalian cells, SERCA is crucial for maintaining low intracellular Ca^2+^ concentrations by sequestering Ca^2+^ within the sarco/endoplasmic reticulum (Arruda & Hotamisligil, 2015). In adult bivalves, SERCA has been suggested to play a role in biomineralization due to its high expression (Truebano et al., 2010) and localized expression of one SERCA isoform (Fan et al., 2007) in mantle tissue. Aside from SERCA, organisms may also employ calcium‐binding proteins to reduce free Ca^2+^ concentrations intracellularly. Expression of contigs for one such calcium‐binding protein, calbindin, was observed to increase during the course of *M. edulis* larval development with a peak in expression at PD I stage. Sodium/calcium exchangers (SLC8) is a group of membrane‐bound transport proteins that facilitate the reversible exchange of three sodium ions (Na^+^) for one calcium ion and has an established role in mammalian osteoblast (bone) calcification and avian eggshell mineralization (Cheidde, Viera, Lima, Saad, & Heilberg, 2003; Sosnoski & Gay, 2007). Recently, immunolabelling techniques have demonstrated that an NCX protein is particularly abundant within calcifying cells of the coral, *Acropora yongei* (Barron et al., 2018). The simultaneous elevated expression profiles of the putative NKA and NCX during *M. edulis* ontogenetic development supported the role of NCX in larval Ca^2+^ transport. Finally, increased transcript abundances for contigs encoding several Ca^2+^ channels (Ca^2+^ load activated Ca^2+^ channel, voltage‐dependent Ca^2+^ channels, Ca^2+^ channel subunit α) were observed during *M. edulis* larval development. Such cellular pathways have also been observed to take part in Ca^2+ ^transport in the calcifying mantle epithelia of adult oysters (Sillanpaa et al., 2018). Specifically, Sillanpaa et al. (2018) suggest the role of NCX proteins on the basolateral membrane, while voltage‐dependent Ca^2+^ channels facilitate Ca^2+^ transport on the apical membranes of the calcifying epithelia in adult oysters, *Crassostrea gigas*.

#### 4.2.3 Na^+^ transport

The Na^+^/K^+^ ATPase (NKA) protein is an active membrane‐bound pump present on the basolateral membrane. It is critical for maintenance of cell membrane potential and generates the electrochemical gradient necessary to facilitate the subsequent transport of ions by secondary transport proteins (Boron & Boulpaep, 2009). The exchange of sodium and hydrogen ions via the sodium/hydrogen exchanger (NHE) belonging to the SLC9 family is one such secondary pathway driven by the NKA. The elevated coexpression patterns of a mitochondrial NHE and NKA, where contigs encoding these proteins exhibit peaks in expression during trochophore development, suggest that the NHE is critical for proton (H^+^) removal (Figure 1u–y). In accordance with the upregulation of contigs encoding NKA during early shell formation, NKA activity has also been recorded to peak during early shell formation in oyster larvae (Frieder, Applebaum, Pan, Hedgecock, & Manahan, 2017). Similar transport processes are present in the primary mesenchymal cells in sea urchin larvae which are responsible for calcification and skeletogenesis where amiloride‐sensitive ion transport proteins such as the NHE have been demonstrated to be significant for cellular pH regulation (Stumpp et al., 2012). In addition to elevated expression of contigs encoding NHEs at the onset of larval calcification, we also observed a peak in expression of gene encoding Na^+^/H^+^ exchange regulatory factor (NHERF). Na^+^/H^+^ exchange regulatory factor proteins are involved in regulating the function of NHE and have a pivotal role in bone formation, where their regulation of NHEs is crucial for osteoblast differentiation and strength (Liu et al., 2012).

#### 4.2.4 H^+^ transport

Apart from NHE (see above), several contigs putatively encoding H^+^ transporters were differentially expressed during larval development. Among these, contigs encoding VHAs exhibited a peak in expression during PD I stage of larval development. In addition to the active transport of H^+^, secondary H^+^ transport pathways such as voltage‐gated hydrogen channels also exhibited dynamic expression profiles during development of *M. edulis*. Proton efflux via voltage‐gated hydrogen channels are responsible for pH homeostasis in calcifying coccolithophore cells, preventing cytoplasmic acidification (Taylor, Chrachri, Wheeler, Goddard, & Brownlee, 2011).The elevated expression of H^+^ transport pathways during larval calcification is consistent with the requirement to extrude protons that are generated by the mineralization of calcium carbonate from HCO_3_^−^ and the observed increases in pH at the site of calcification in larval mussels (Ramesh et al., 2017).

#### 4.2.5 Transport of other ions

Parallel to the acquisition of substrates for calcification (Ca^2+^ and HCO_3_^−^) and removal of proton by‐products of calcification, there are ion transport proteins that are essential for maintenance of cellular electrogenic gradients, cell volume, etc. During the ontogenetic development of *M. edulis*, expression of several contigs encoding chloride (Cl^−^) and potassium (K^+^) channels was observed. In particular, elevated expression for various chloride channels during PD I larval stage was detected (Figure S3). Efflux of Cl^−^ from the basolateral membrane due to acquisition of HCO_3_^−^ in the calcification space via anion exchangers may be coupled to the elevated activity of Cl^−^ channels. Alternatively, Cl^−^ efflux may occur via cation‐coupled pathways (SLC12) or H^+^/Cl^−^ exchange. However, contigs encoding such Cl^−^ transport proteins did not exhibit high transcript abundances during larval development or shell formation in *M. edulis*.

Expression of several K^+^ channels was seen in the larval transcriptome. However, significant changes in the expression of only one K^+^ channel was observed during the course of development, the inward rectifier K^+^ channel (K_ir_, Figure S3). Interestingly, this group of membrane‐bound transport proteins was also found to be upregulated during shell repair in adult *M. edulis* (Yarra, 2018). K_ir_ channels are important in sustaining electrochemical gradients and cell resting potential by recycling K^+^ ions (Weber, Cunningham, & Schulte, 2001). In addition, their absence in mammalian osteoblasts has been observed to inhibit osteoblastgenesis due to a decreased efficiency in production of an extracellular matrix (Sacco et al., 2015).

### 4.3 Shell matrix proteins (SMPs)

It has long been known that SMPs play a critical role in calcium deposition and shell development (Weiner & Traub, 1984; Wheeler & Sikes, 1984), but these are poorly characterized in larvae. Interestingly, approximately, 65% of the SMPs expressed in the adult Baltic *M. edulis*‐like mantle transcriptome (Yarra, 2018) and ca. 65% of the SMPs extracted from adult shells were expressed during larval development. Similar to *M. edulis*‐like larvae, three SMPs (nacrein, EGF‐like, and tyrosinase) have also been observed to be expressed in other bivalve larvae (Fang et al., 2011; Li, Zhang, et al., 2016; Liu et al., 2015). Multiple contigs with sequence similarity to other adult shell SMPs, but not yet identified in larval shells, were found to be differentially expressed throughout PD I development. Most of the differentially expressed contigs encoded domains involved in structuring the shell or tissue, such as β‐hexosaminidase, glycoside hydrolase, chitin synthase, chitin binding, von Willebrand factor A, and Fibronectin type III. These were all found to have an increasing expression profile as the shell field was expanding over the surface of the larvae. Contigs containing copper‐binding domains such as amine oxidase, dopamine‐β‐hydroxylase, and tyrosinase were also shown to increase in expression as the PD I shell was formed. Of particular interest were contigs containing tyrosinase domains that showed a decrease in expression right before the end of PD I formation. Tyrosinase proteins are involved in periostracum formation (Zhang, Xie, Huang, Chen, & Zhang, 2006), and the drop in expression of contigs containing tyrosinase domains may reflect the completion of periostracum formation in PD I.

Other SMPs linked to mineral deposition and crystallographic control include proteases and protease inhibitors that behave antagonistically, where the former are known to be important for crystal nucleation (Hershey et al., 2016; Tiaden et al., 2012). During the formation of the Baltic *M. edulis*‐like PD I shell, contigs containing protease inhibitor domains such as BPTI/Kunitz and Kazal were increasing in expression throughout shell formation. BPTI/Kunitz domain‐containing contigs exhibited a peak in expression prior to the completion of PD I, supporting the domains involvement in terminating crystal growth. Conversely, the contig with the protease inhibitor domain β‐lactamase (Gigasin 6) was highly expressed before larval calcification, suggesting a putative involvement in initial crystal deposition. The contig containing the protease domain, peptidase C1A, was observed to increase in expression throughout shell formation, thus not following the pattern of other protease and protease inhibitors. However, this may be because peptidase C1A domains occur in multifunctional proteins which are also involved in immune functions.

Although bivalve larval shells are composed of only one calcium carbonate polymorph, aragonite (Kudo et al., 2010; Yokoo et al., 2011), several SMPs (tyrosinase, gigasin‐like and alveoline‐like proteins) that were previously identified in the calcitic fibrous prism structures of adult mytilid shells (Gao et al., 2015; Liao et al., 2015) were also expressed in the Baltic *M. edulis*‐like developing larvae. This emphasizes the need for further investigation into larval stages over a longer developmental time period to determine whether there is specific SMP partitioning with development.

## 5 CONCLUSIONS

By rearing mussel larvae under conditions of substrate limitation for calcification and analyzing differential gene expression patterns, we were able to identify a membrane‐bound transport protein potentially involved in HCO_3_^−^ acquisition, belonging to the SLC26 family of anion transporters and other candidate genes previously identified in human biomineralization. Interestingly, the present study identifies only a small subset of contigs to be differentially expressed under substrate limitation. Although this may be improved by a stronger *C*_T_ treatment (>50% decrease relative to control), higher sequencing depth, and level of replication, bivalve larvae may possess a fixed capacity to modify their transcriptomic developmental program. This is consistent with previous studies on bivalve larval development that observed no significant changes in gene expression in response to induced acid–base stress (Kelly et al., 2016). Our data demonstrated an increased expression of contigs encoding for Ca^2+^ and HCO_3_^−^‐transporting proteins during larval development, in particular, once larval shell formation started. In particular, the dynamic expression patterns and high expression levels of contigs encoding SERCA, NCX, and NBC hint toward the role of these ion transport pathways in bivalve larval calcification. Similarly, the analyses of SMP expression patterns revealed several proteins with hypothesized roles in shell structure, crystallographic control, and periostracum deposition to be upregulated during larval development. To date, functional analyses using RNA interference techniques have been limited to SMPs (Fang et al., 2011; Funabara et al., 2014; Suzuki et al., 2009). However, knock out/knock down techniques are required to establish the role of candidate ion transporters in larval calcification. The identification of candidate biomineralization genes in this study paves the way for future in depth investigations. Heterologous expression techniques in conjunction with electrophysiology techniques should be used to characterize the substrate specificity and stoichiometry of these ion transport genes (in particular, the SLC26A11 ortholog) and their complex interactions with SMPs to produce a robust larval shell.

## CONFLICT OF INTEREST

None declared.

## AUTHOR CONTRIBUTIONS

K.R. and F.M. designed the study. K.R. conducted the larval experiments, RNA extractions, and sequencing library preparations. T.Y conducted the bioinformatics analysis. K.R. and T.Y. analyzed the data and wrote the manuscript. All authors contributed to paper revisions.

## Data Availability

Transcriptomics data are available at the NCBI short read archive under accession number PRJNA494236.
