# Supplementary material for: Expression of calcification‐related ion transporters during blue mussel larval development
Source: Ecol Evol. 2019 May 29;9(12):7157–72. doi: 10.1002/ece3.5287 (PMC6662379; doi:10.1002/ece3.5287)
Supplement: Supplementary file 1 [file ECE3-9-7157-s001.pdf]

Supplementary Methods

Correcting library names

After initial data processing, some of the libraries appeared to be mislabeled. The possible library mislabeling was investigated and corrected for, by aligning the cleaned reads to a mitochondrian sequence, and clustering based on variants.

Single nucleotide polymorphism (SNP) calling pipeline

Cleaned libraries were aligned to a M. edulis mitochondrion sequence (Accession: NC 006161.1) using bwa mem (Li and Durbin 2009) with the -M flag. Output from the alignment was sorted using samtools (sort) (Li et al 2009), and Picard tools (Broad Institute, http://broadinstitute.github.io/ picard) were used to mark and remove duplicates (MarkDuplicates) and realign the alignment file (AddOrReplaceRead- Groups). All libraries were grouped based on calculated genotype likelihood using samtools (mpileup), and VarScan-2 (Kobolt et al 2012) was used to detect variants. Variants were filtered based on a density level of 5 using scripts published as part of SNP calling pipeline (Quick 2014). Filtered variants file was converted to a sequence based fasta file using a published script (Loman 2015), which was then converted to newick format using FastTree-2 (Price 2010), for viewing of the phylogenetic tree in FigTree (http://tree.bio.ed.ac.uk/software/figtree/).

Uncorrected multidimensional scaling (MDS) plots of the digital expression levels showed a clear separation among developmental stages and each of the larval families (Supplementary Figure S4). However, two of the sequenced libraries appeared to be misnamed. Treated libraries in family 003 and family 004 appeared to be mixed up in stage 2, and treated libraries at stage 5 and stage 6 appeared to be mixed up in family 004. Clustering SNPs identified by aligning all libraries to a published mitochondrion sequence (Supplementary Figure S5) confirmed that the mixup in treated libraries in stage 2 between family 003 and family 004 was due to mislabeled files and not due to biological reasons. Such a validation was not possible for the mixup in family 004 between stage 5 and stage 6, but given the clear expression trends all other libraries, the mislabeling was 'fixed' for further analysis (Supplementary Figure S6).

Supplementary References

Li H, Durbin R (2009) Fast and accurate short read alignment with Burrows-Wheeler transform. Bioinformatics 25.14, Pages 1754–1760.

Li H, Handsaker B, Wysoker A, Fennell T, Ruan J, Homer N, Marth G, Abecasis G, Durbin R (2009) The Sequence Alignment/Map format and SAMtools. Bioinformatics 25.16 Pages 2078–2079.

Koboldt DC, Zhang Q, Larson DE, Shen D, McLellan MD, Lin L, Miller CA, Mardis ER, Ding L, Wilson RK (2012) VarScan 2: somatic mutation and copy number alteration discovery in cancer by exome sequencing. Genome Res. 22.3 Pages 568– 576.

Quick J (2014) SNP calling pipeline. url: https://github.com/joshquick/snp_ calling_scripts

Loman N (2015) Misc. genomic tools. url: https://github.com/nickloman/misc-genomics-tools/tree/master/scripts.

Price MN, Dehal PS, Arkin AP (2010) FastTree 2–approximately maximum- likelihood trees for large alignments. PLoS ONE 5.3, e9490.

Supplementary Figure Legends

Supplementary Figure S1 | The morphologically distinct larval developmental stages sampled during the experiments. (a-b) Pre-calcification (c-d) Onset of calcification and larvae exhibit dorsal flattening at the region of the shell field, (e-f) Early trochophore shell covering ca 10% of larval body is observed by the presence of a small (ca. 20 µm) shell and birefringence (dependent on larval orientation) at the hinge area, (g-h) ca. 49% of the larval body is covered by a mineralized shell, (i-j) ca. 76% of the larval body is covered by a mineralized shell, (k-l) Larvae have secreted the PD I shell and exhibit a distinctive 'D' shape. Scale bars are 20 µm.

Supplementary Figure S2 | Mean expression fold change of the 53 genes differentially expressed between control and low C_T libraries.

Supplementary Figure S3 | Genes encoding K^+ and Cl^- transport proteins differentially expressed during the course of larval development in *M. edulis* reared under ambient conditions. Expression values (normalized read counts) by EBSeqHMM for (a-b) K^+ channels: Inward rectifier K^+ channels (c-e) Cl^- channels. Maximum posterior probability for all contigs are reported in Supplementary Table S7.

Supplementary Figure 4 | MDS plot of expression counts from filtered libraries. Boxes show possible misnamed libraries.

Supplementary Figure 5 | Clustering of SNPs in family 003 and 004 based on alignment to reference *M. edulis* mitochondrion sequence (Accession: NC 006161.1), C - Control, T – Treated (low C_T).

Supplementary Figure 6 | MDS plot of expression counts from filtered data after adjusting misnamed libraries.

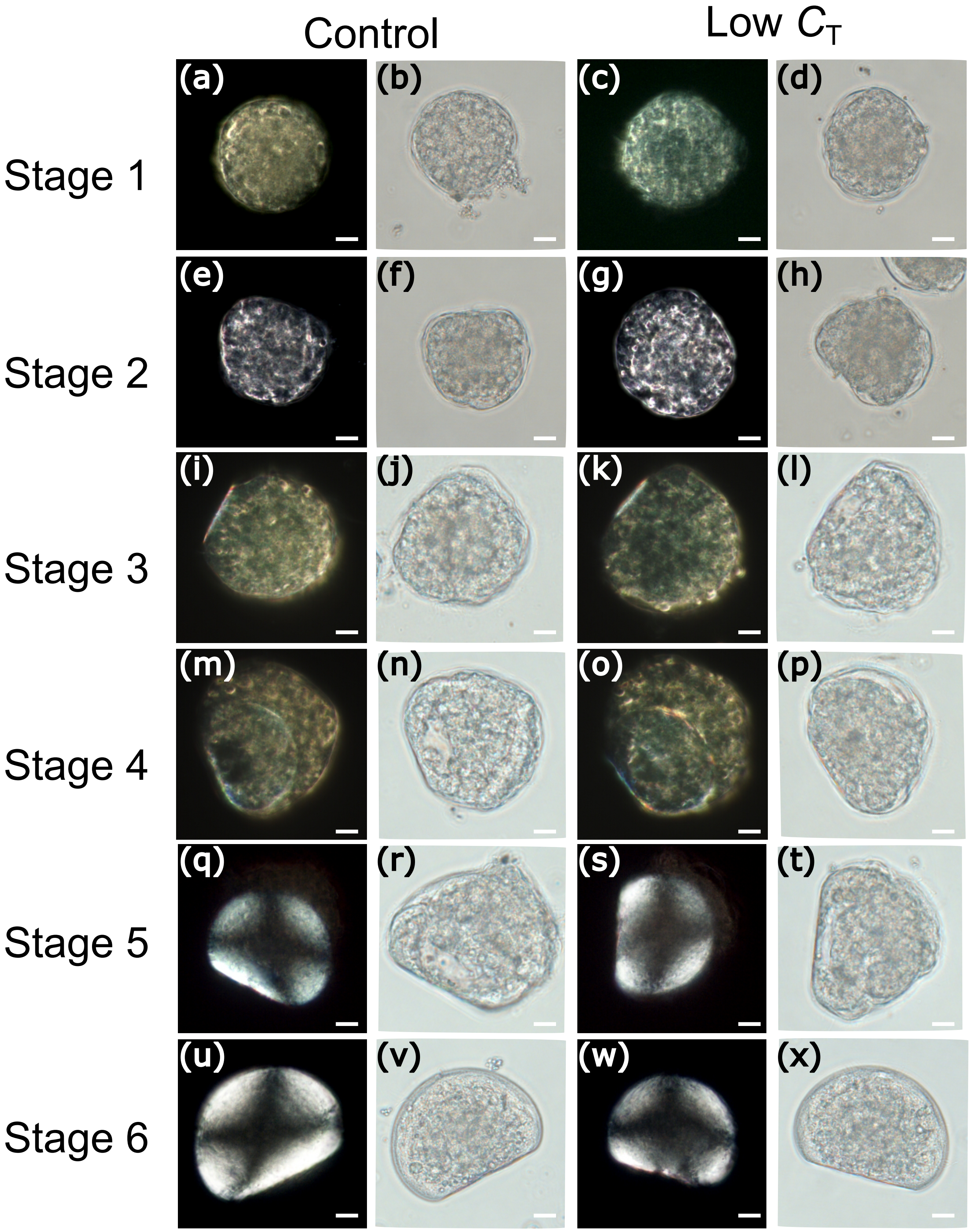
Control
Low C_T
Stage 1
Stage 2
Stage 3
Stage 4
Stage 5
Stage 6
(a)
(b)
(c)
(d)
(e)
(f)
(g)
(h)
(i)
(j)
(k)
(l)
(m)
(n)
(o)
(p)
(q)
(r)
(s)
(t)
(u)
(v)
(w)
(x)

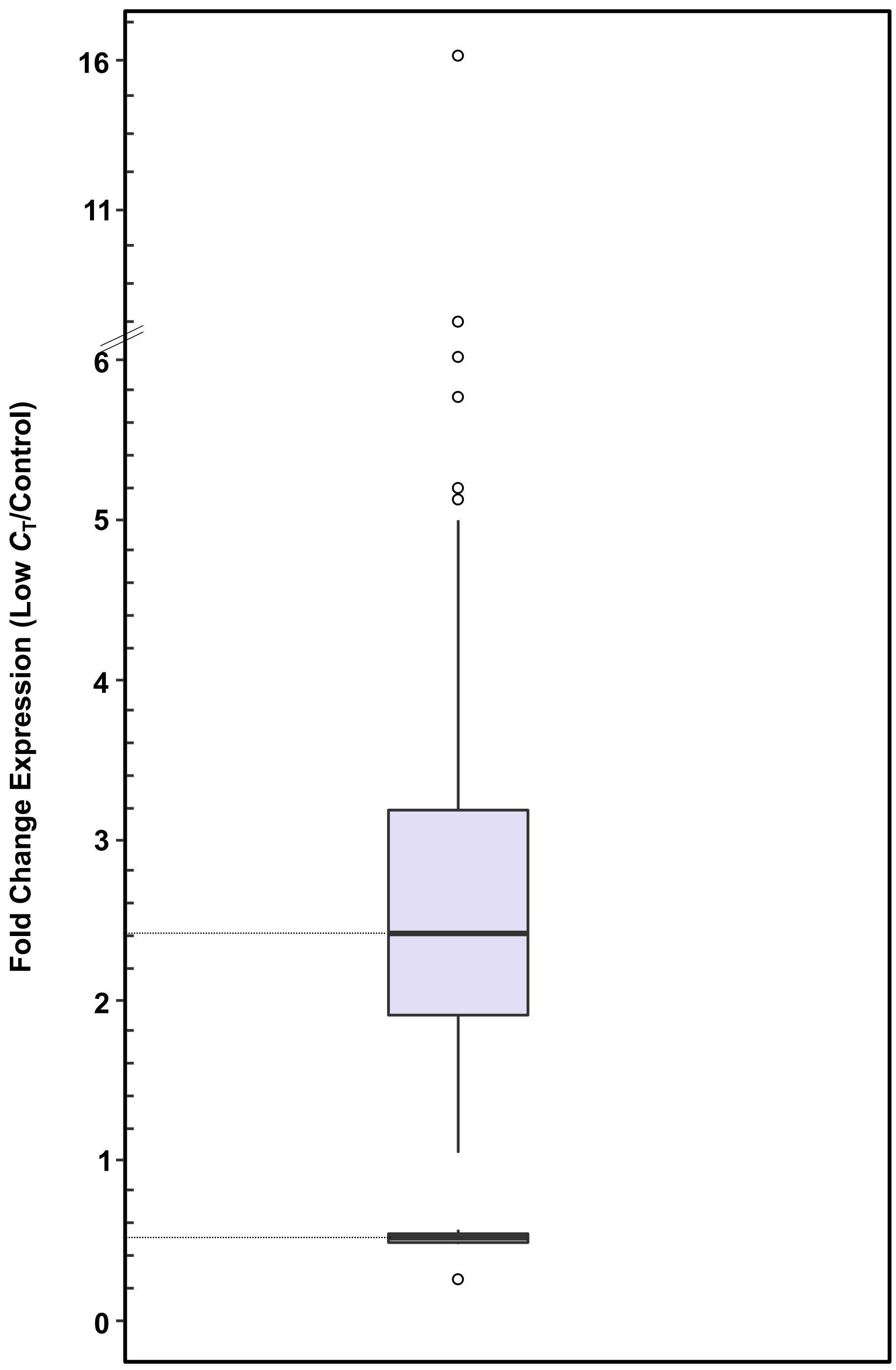
Fold Change Expression (Low C_T/Control)
16
11
6
5
4
3
2
1
0

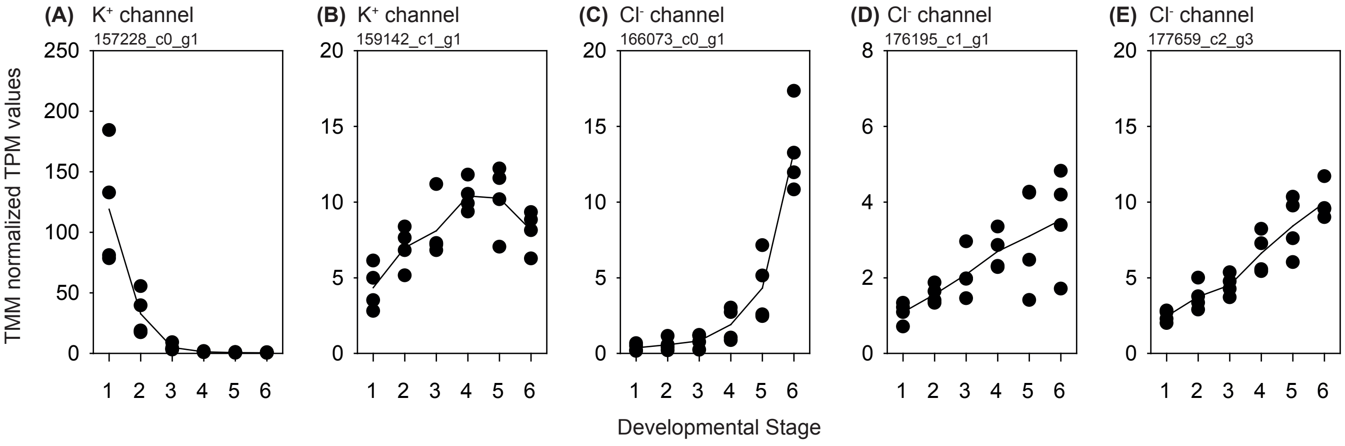

(A) K+ channel
157228_c0_g1
(B) K+ channel
159142_c1_g1
(C) Cl- channel
166073_c0_g1
(D) Cl- channel
176195_c1_g1
(E) Cl- channel
177659_c2_g3
TMM normalized TPM values
Developmental Stage

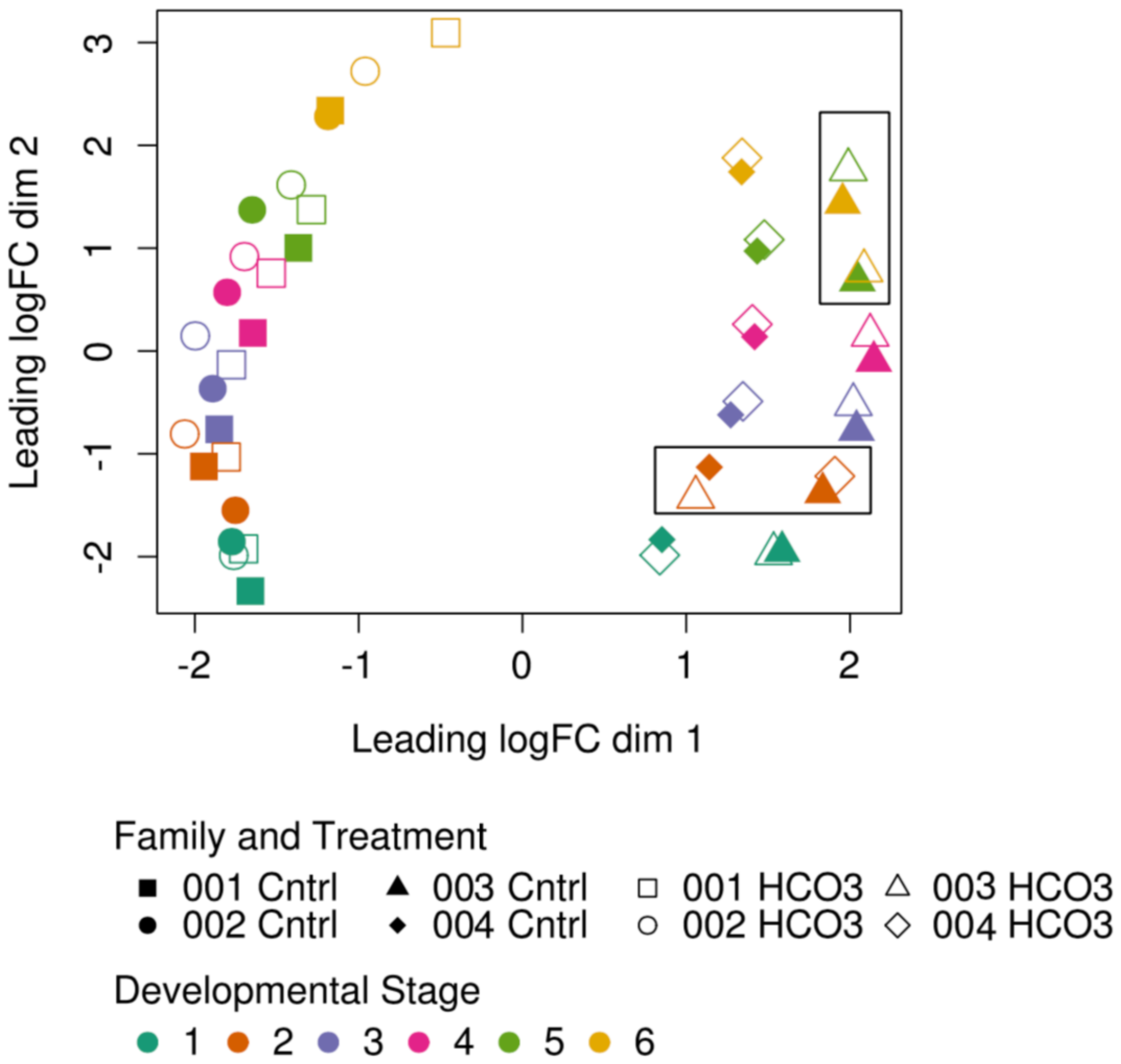
Leading logFC dim 2
Leading logFC dim 1
3
2
1
0
-1
-2
-2
-1
0
1
2
Family and Treatment
001 Cntrl
002 Cntrl
003 Cntrl
004 Cntrl
001 HCO3
002 HCO3
003 HCO3
004 HCO3
Developmental Stage
1
2
3
4
5
6

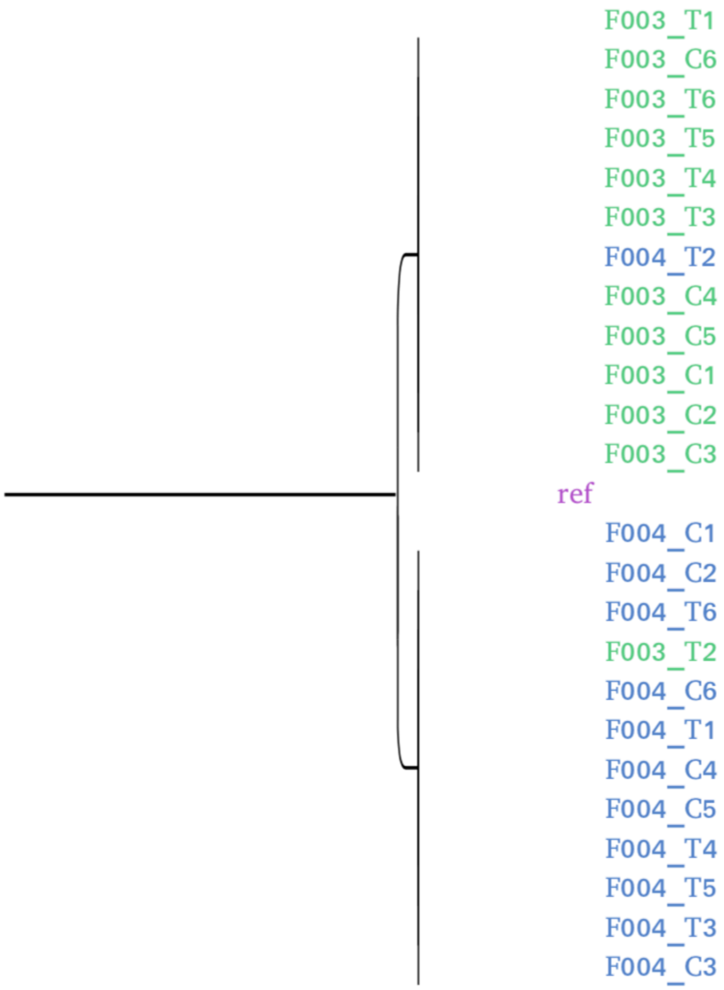
F003_T1
F003_C6
F003_T6
F003_T5
F003_T4
F003_T3
F004_T2
F003_C4
F003_C5
F003_C1
F003_C2
F003_C3
ref
F004_C1
F004_C2
F004_T6
F003_T2
F004_C6
F004_T1
F004_C4
F004_C5
F004_T4
F004_T5
F004_T3
F004_C3

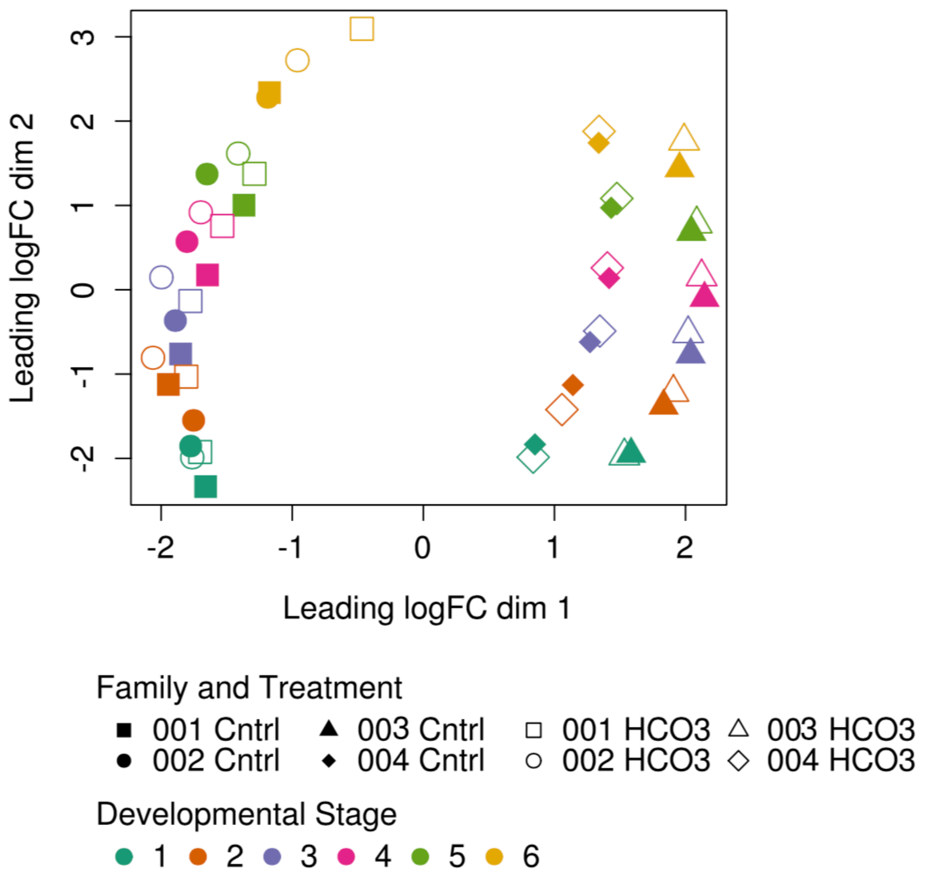

Leading logFC dim 2
Leading logFC dim 1
-2
-1
0
1
2
3
Family and Treatment
001 Cntrl
002 Cntrl
003 Cntrl
004 Cntrl
001 HCO3
002 HCO3
003 HCO3
004 HCO3
Developmental Stage
1
2
3
4
5
6

Supplementary Table S1. Seawater carbonate chemistry parameters of the experiments. Parameters of CO_2, HCO_3^- and CO_3^{2-} are calculated from measured C_T and pH_{NBS} using CO2SYS.

Experiment	Treatment	Salinity	Temperature (°C)	pH_{NBS}	C_T (µmol kg^{-1} seawater)	CO_2 (µmol/kg^{-1} seawater)	[HCO_3^-] (µmol kg^{-1} seawater)	[CO_3^{2-}] (µmol kg^{-1} seawater)	pCO_2 (uatm)	$\Omega_{aragonite}$
1	Control	15.3	17	8.211	1959.08	17.01	1837.62	104.44	433.81	1.67
1	C_T limited	15.3	17	8.12	879.83	9.49	831.98	38.34	242.19	0.61
2	Control	15.1	17	8.225	1942.27	16.37	1819.92	105.977	417.11	1.70
2	C_T limited	15.1	17	8.161	919.13	9.03	866.55	43.54	230.14	0.69
3	Control	16.1	17	8.217	1957.62	16.47	1832.14	108.99	422.09	1.74
3	C_T limited	16.1	17	8.104	987.03	10.88	933.33	42.80	278.92	0.68
4	Control	15.6	17	8.231	2003.24	16.47	1873.89	112.87	420.78	1.811
4	C_T limited	15.6	17	8.19	980.86	8.90	921.45	50.50	227.39	0.81

Supplementary Table S2. Accession ID's for protein sequences used in the phylogenetic tree in Figure 8.

	Accession ID	Accession Source
**Solute carrier family 4:**		
Stylophora pistillata SLC4-alpha	KJ829357	NCBI
Stylophora pistillata SLC4-beta	KJ829358	NCBI
Stylophora pistillata SLC4-gamma	KJ829359	NCBI
Stylophora pistillata SLC4-delta	KJ829360	NCBI
Stylophora pistillata SLC4-epsilon	KJ829361	NCBI
Homo sapiens SLC4A1	B3AT_HUMAN	Uniprot
Homo sapiens SLC4A2	B3A2_HUMAN	Uniprot
Homo sapiens SLC4A3	B3A3_HUMAN	Uniprot
Homo sapiens SLC4A4	S4A4_HUMAN	Uniprot
Homo sapiens SLC4A5	S4A5_HUMAN	Uniprot
Homo sapiens SLC4A7	S4A7_HUMAN	Uniprot
Homo sapiens SLC4A8	S4A8_HUMAN	Uniprot
Homo sapiens SLC4A9	B3A4_HUMAN	Uniprot
Homo sapiens SLC4A10	S4A10_HUMAN	Uniprot
Crassostrea gigas AE-2	EKC18228.1	NCBI
Crassostrea gigas SLC4-1	EKC36355.1	NCBI
Crassostrea gigas SLC4-2	EKC35200.1	NCBI
Strongylocentrotus purpuratus NBC2	SPU_025514	EchinoBase
**Solute carrier family 26:**		
Stylophora pistillata SLC26-alpha	KJ829362	NCBI
Stylophora pistillata SLC26-beta	KJ829363	NCBI
Stylophora pistillata SLC26-gamma	KJ829364	NCBI

Homo sapiens SLC26A1	S26A1_HUMAN	Uniprot
Homo sapiens SLC26A2	S26A2_HUMAN	Uniprot
Homo sapiens SLC26A3	S26A3_HUMAN	Uniprot
Homo sapiens SLC26A4	S26A4_HUMAN	Uniprot
Homo sapiens SLC26A5	S26A5_HUMAN	Uniprot
Homo sapiens SLC26A6	S26A6_HUMAN	Uniprot
Homo sapiens SLC26A7	S26A7_HUMAN	Uniprot
Homo sapiens SLC26A8	S26A8_HUMAN	Uniprot
Homo sapiens SLC26A9	S26A9_HUMAN	Uniprot
Homo sapiens SLC26A10	S2610_HUMAN	Uniprot
Homo sapiens SLC26A11	S2611_HUMAN	Uniprot
**Outlier:**		
Danio rerio CFTR	CFTR_DANRE	Uniprot

Supplementary Table S3. Annotations of differentially expressed contigs in treatment libraries compared to control libraries. Up and down-regulation is marked by '+' and '-' respectively.

Contig ID	Annotation	Accession ID	Developmental Stage	FDR (log_{10})	Fold Change
TRINITY_DN162334_c0_g3			3, 4, 5	$5.48E^{-15}$, $9.74E^{-20}$, $1.54E^{-11}$,	+5.77, +4.95, +3.16
TRINITY_DN164559_c3_g2	TNFAIP3 interacting protein 2 (*Homo sapiens*)	TNIP2_HUMAN	3, 4, 5, 6	$2.43E^{-11}$, $3.44E^{-11}$, $5.77E^{-10}$, $2.40E^{-05}$	+2.62, +2.53, +2.41, +2.02
TRINITY_DN161315_c0_g1	Cdc42 homolog (*Drosophila melanogaster*)	CDC42_DROME	3, 4	$1.69E^{-09}$, $2.24E^{-05}$	+3.18, +2.42
TRINITY_DN175794_c1_g2	Interleukin 1 receptor associated kinase 4 (*Homo sapiens*)	IRAK4_HUMAN	3, 5	$3.31E^{-09}$, $8.09E^{-05}$	+2.09, +1.75
TRINITY_DN157412_c0_g1	NF kappa B inhibitor α (*Gallus gallus*)	IKBA_CHICK	3, 4, 5, 6	$4.37E^{-07}$, $4.98E^{-06}$, $1.46E^{-06}$, $1.01E^{-04}$	+1.91, +1.82, +1.86, +1.74
TRINITY_DN176188_c0_g5	Nuclear transcription factor Y subunit γ (*Pongo abelii*)	NFYC_PONAB	3, 4, 5	$1.32E^{-06}$, $2.38E^{-07}$, $4.63E^{-07}$	+2.15, +2.21, +2.18
TRINITY_DN164091_c3_g1	Excitatory amino acid transporter 3 (*Mus musculus*)	EAA3_MOUSE	3, 4	$6.89E^{-06}$, $1.26E^{-07}$	+2.47, +2.65
TRINITY_DN154143_c0_g2	Putative uncharacterized transposon derived protein F54H12.3	YMD3_CAEEL	3	$1.65E^{-05}$,	+2.98

	(*Caenorhabditis elegans*)				
TRINITY_DN164725_c0_g5			3, 4, 5, 6	$2.46E^{-05}$, $5.28E^{-05}$, $2.32E^{-06}$, $4.57E^{-04}$	+2.32, +2.26, +2.47, +2.11
TRINITY_DN154643_c1_g1	Uncharacterized protein (*Crassostrea gigas*)	K1RHH1_CRAGI	3	$2.46E^{-05}$,	+3.55
TRINITY_DN156025_c1_g3	Runt related transcription factor 1 (*Homo sapiens*)	RUNX1_HUMAN	3, 5	$6.10E^{-05}$, $3.84E^{-04}$	+2.02, +1.91
TRINITY_DN175161_c0_g6			3, 5	$1.61E^{-04}$, $2.32E^{-06}$	+5.13, +4
TRINITY_DN138179_c1_g3			3	$1.65 E^{-04}$,	+4.46
TRINITY_DN152071_c1_g3	Uncharacterized protein (*Tetranychus urticae*)	A0A158P512_TETUR	3	$3.59 E^{-04}$,	+2.8
TRINITY_DN25158_c0_g1			3	$5.32 E^{-04}$,	+2.08
TRINITY_DN149718_c0_g9			3	$8.42 E^{-04}$,	+6.02
TRINITY_DN160667_c0_g2	Failed axon connections homolog (*Homo sapiens*)	FAXC_HUMAN	4	$2.69E^{-10}$	-0.52
TRINITY_DN175059_c1_g4	Sodium-independent sulfate anion	S2611_MOUSE	4, 5	$8.55E^{-07}$, $1.34E^{-12}$	+2.32, +2.96

	transporter (*Mus musculus*)				
TRINITY_DN169005_c1_g4	ETS related transcription factor Elf 3 (*Crassostrea gigas*)	K1QAA0_CRAGI	4, 5, 6	6.45E^{-06}, 4.01E^{-10}, 2.08E^{-08}	+1.82, +2.14, +2.05
TRINITY_DN170535_c0_g2			4, 5	6.76E^{-05}, 1.94E^{-04}	+3.16, +2.94
TRINITY_DN168878_c0_g1	α-glucosidase yihQ (*Crassostrea gigas*)	K1QZH8_CRAGI	4	7.34E^{-05}	+1.9
TRINITY_DN153040_c0_g1	Uncharacterized protein (*Lottia gigantea*)	V3Z9W2_LOTGI	4	7.19E^{-04}	+1.55
TRINITY_DN143945_c0_g1			4	8.29E^{-04}	+2.46
TRINITY_DN158062_c0_g1	Transcription factor Sox 8 (*Xenopus laevis*)	SOX8_XENLA	5, 6	6.63E^{-10}, 3.05E^{-11}	+2.78, +3.41
TRINITY_DN165794_c0_g8	Adipocyte plasma membrane associated protein (*Bos taurus*)	APMAP_BOVIN	5, 6	3.68E^{-06}, 6.04E^{-04}	-0.5, -0.48
TRINITY_DN85247_c0_g1			5, 6	1.14E^{-05}, 8.85E^{-06}	+3.24, +3.41
TRINITY_DN177115_c1_g1	Tumor necrosis factor alpha induced protein 3 (*Mus musculus*)	TNAP3_MOUSE	5	1.71E^{-05}	+1.9

TRINITY_DN178440_c1_g3	Nuclear factor NF kappa B p105 subunit (*Rattus norvegicus*)	NFKB1_RAT	5	2.96E^{-05}	+1.54
TRINITY_DN164963_c0_g1			5	3.52E^{-05}	+1.6
TRINITY_DN153976_c2_g1	Nose resistant t fluoxetine protein 6 (*Caenorhabditis elegans*)	NRF6_CAEE	5	8.09E^{-05}	+1.59
TRINITY_DN176455_c1_g2			5	2.90E^{-04}	+2.23
TRINITY_DN158676_c0_g1	Ras related C3 botulinum toxin substrate 1 (*Rattus norvegicus*)	RAC1_RAT	5	7.10E^{-04}	+2.36
TRINITY_DN162758_c0_g2	Endoglucanase E 4 (*Thermobifida fusca*)	GUN4_THEFU	5	7.41E^{-04}	+1.61
TRINITY_DN160914_c0_g1	Putative Ankyrin repeat protein MM 0045 (*Methanosarcina mazei*)	Y045_METMA	5, 6	7.41E^{-04}, 3.46E^{-05}	+2.77, +3.31
TRINITY_DN178074_c2_g4	Zinc transporter ZIP13 (*Danio rerio*)	S39AD_DANRE	5	8.14E^{-04}	+2.41
TRINITY_DN166023_c2_g1	Uncharacterized protein (*Arion vulgaris*)	A0A0B6ZCF8_9EUPU	6	1.14E^{-05}	+3.7
TRINITY_DN160914_c0_g2			6	6.40E^{-05}	+3.2

TRINITY_DN160789_c2_g2			6	8.93E^{-05}	+1.74
TRINITY_DN144435_c0_g1	Putative C-type lectin (*Haliotis discus hannai*)	A0A0D3MJB9_HALDH	6	1.77E^{-04}	+16.11
TRINITY_DN159064_c3_g3			6	1.83E^{-04}	+7.16
TRINITY_DN180876_c0_g1	Retrovirus related Pol polyprotein from transposon 297 (*Drosophila melanogaster*)	POL2_DROME	6	2.14E^{-04}	-0.57
TRINITY_DN168079_c0_g2	Serine protease inhibitor dipetalogastin (*Crassostrea gigas*)	K1PRF2_CRAG	6	2.27E^{-04}	-0.26
TRINITY_DN83729_c0_g1	Mammalian ependymin related protein 1 (*Homo sapiens*)	EPDR1_HUMAN	6	4.82E^{-04}	+2.6
TRINITY_DN169769_c2_g1	Heme-binding protein 2 (*Homo sapiens*)	HEBP2_HUMAN	6	4.82E^{-04}	+2.62
TRINITY_DN147142_c0_g1	DBH like monooxygenase protein 1 homolog (*Danio rerio*)	MOXD1_DANRE	6	4.82E^{-04}	+5.2
TRINITY_DN173237_c0_g2	Retinal dehydrogenase 2 (RALDH)(*Mus musculus*)	AL1A2_MOUSE	6	4.82E^{-04}	+1.69

TRINITY_DN137986_c0_g1	Expansin yoaJ (*Crassostrea gigas*)	K1QR45_CRAGI	6	4.84E^{-04}	+4.59
TRINITY_DN183378_c0_g1	Uncharacterized protein (*Lottia gigantea*)	V4BE89_LOTGI	6	5.07E^{-04}	+1.82
TRINITY_DN148276_c0_g1			6	5.32E^{-04}	+3.7
TRINITY_DN176745_c2_g2	DEP domain containing protein 1A (*Danio rerio*)	DEP1A_DANRE	6	5.34E^{-04}	-0.52
TRINITY_DN157627_c0_g2			6	5.34E^{-04}	+3.63
TRINITY_DN180865_c8_g1	Uncharacterized protein (*Crassostrea gigas*)	K1PTU7_CRAGI	6	6.04E^{-04}	-0.57
TRINITY_DN176739_c2_g1	Malate dehydrogenase (*Pyrococcus abyssi*)	MDH_PYRAB	6	6.15E^{-04}	+1.84

Supplementary Table S4. Number of differentially expressed contigs between developmental stages in both control and treated libraries.

	Number of differentially expressed contigs in control group	Number of differentially expressed contigs in treatment group
**Stage 1 as baseline:**		
Stage 2 vs.	228	369
Stage 1	-17	-70
Stage 3 vs.	1,104	1,763
Stage 1	-590	-1,047
Stage 4 vs.	2,588	2,775
Stage 1	-1,830	-2,282
Stage 5 vs.	3,677	3,723
Stage 1	-3,269	-3,535
Stage 6 vs.	4,838	5,868
Stage 1	-4,423	-5,544
**Consecutive stages:**		
Stage 2 vs.	228	369
Stage 1	-17	-70
Stage 3 vs.	87	283
Stage 2	-60	-135
Stage 4 vs.	246	168
Stage 3	-85	-173
Stage 5 vs.	174	50
Stage 4	-120	-43
Stage 6 vs.	395	1,417
Stage 5	-589	-1,278

Supplementary Table S5. Annotations of differentially expressed contigs in larvae at Stage 2 (onset of calcification) compared to Stage 1 (pre-calcification).

Contig ID	Annotation	Accession ID	FDR
TRINITY_DN119635_c0_g1			$1.49E^{-04}$
TRINITY_DN156475_c0_g1	Forkhead box protein L2 (*Crassostrea gigas*)	K1RVN0_CRAGI	$4.83E^{-06}$
TRINITY_DN135592_c0_g1			$1.68E^{-04}$
TRINITY_DN135592_c0_g2			$5.63E^{-06}$
TRINITY_DN30478_c0_g1			$7.23E^{-04}$
TRINITY_DN162074_c1_g3	GTP-binding protein GEM (*Homo sapiens*)	GEM_HUMAN	$4.89E^{-04}$
TRINITY_DN146390_c1_g2			$6.70E^{-05}$
TRINITY_DN93876_c0_g2			$3.25E^{-04}$
TRINITY_DN48980_c0_g1	Uncharacterized protein (*Crassostrea gigas*)	K1QY92_CRAGI	$4.33E^{-05}$
TRINITY_DN157509_c0_g5	Zinc transporter ZIP1 (*Bos taurus*)	S39A1_BOVIN	$1.07E^{-08}$
TRINITY_DN160567_c0_g2			$1.08E^{-04}$
TRINITY_DN146390_c0_g1			$1.38E^{-04}$
TRINITY_DN99979_c0_g1			$1.07E^{-08}$
TRINITY_DN17787_c0_g1			$1.50E^{-09}$
TRINITY_DN143936_c0_g1			$2.36E^{-04}$
TRINITY_DN160539_c4_g9			$1.85E^{-04}$
TRINITY_DN161972_c1_g1			$1.02E^{-07}$
TRINITY_DN153405_c3_g4			$6.78E^{-04}$
TRINITY_DN117209_c0_g1			$1.33E^{-06}$
TRINITY_DN167796_c1_g1			$2.77E^{-04}$

TRINITY_DN51677_c0_g1			$1.78E^{-04}$
TRINITY_DN218341_c0_g1			$1.17E^{-06}$
TRINITY_DN57015_c0_g1			$2.09E^{-04}$
TRINITY_DN106379_c0_g1	Uncharacterized protein (*Crassostrea gigas*)	K1S2E8_CRAG	$8.75E^{-05}$
TRINITY_DN128743_c0_g1			$1.65E^{-06}$
TRINITY_DN149108_c0_g1			$7.25E^{-06}$
TRINITY_DN163813_c0_g1	Parathyroid hormone/Parathyroid hormone related peptide receptor (*Didelphis virginiana*)	PTH1R_DIDVI	$4.68E^{-05}$
TRINITY_DN185973_c0_g1			$3.88E^{-05}$
TRINITY_DN169595_c0_g2	Protein unc 93 homolog A (*Xenopus laevis*)	UN93A_XENLA	$4.95E^{-06}$
TRINITY_DN160411_c0_g2	Lactase phlorizin hydrolase (*Homo sapiens*)	LPH_HUMAN	$1.17E^{-06}$
TRINITY_DN64699_c0_g2			$1.12E^{-05}$
TRINITY_DN169461_c0_g2	Major egg antigen (*Schistosoma mansoni*)	P40_SCHMA	$6.53E^{-06}$
TRINITY_DN17787_c0_g2			$2.17E^{-04}$
TRINITY_DN43222_c0_g1	Ferric chelate reductase 1 (*Bathymodiolus platifrons*)	A0A100XKJ2_9BIVA	$1.91E^{-04}$
TRINITY_DN133286_c0_g1			$1.37E^{-06}$

TRINITY_DN161508_c0_g1	EF hand domain containing protein D1 (*Crassostrea gigas*)	K1QRD1_CRAGI	$2.59E^{-07}$
TRINITY_DN144535_c0_g1			$2.41E^{-05}$
TRINITY_DN172433_c0_g1	KS-rich protein (*Mytilus coruscus*)	A0A0K0YB04_MYTCO	$1.36E^{-05}$
TRINITY_DN157573_c0_g1			$1.31E^{-06}$
TRINITY_DN134345_c0_g1			$1.12E^{-05}$
TRINITY_DN145763_c0_g1	Meprin A subunit α (*Homo sapiens*)	MEP1A_HUMAN	$7.73E^{-08}$
TRINITY_DN170726_c0_g4			$1.26E^{-04}$
TRINITY_DN145450_c0_g1	Metalloendopeptidase (*Crassostrea gigas*)	K1S6S4_CRAGI	$1.95E^{-05}$
TRINITY_DN168864_c0_g1	Uncharacterized protein (*Capitella teleta*)	R7UWJ8_CAPTE	$5.57E^{-05}$
TRINITY_DN162493_c0_g3	Uncharacterized protein (*Crassostrea_gigas*)	K1QCE7_CRAGI	$2.99E^{-04}$
TRINITY_DN170889_c0_g5			$5.30E^{-04}$
TRINITY_DN53458_c0_g1	Phosphatidylethanolamine-binding protein homolog F40A3.3 (*Caenorhabditis elegans*)	PEBPH_CAEEL	$1.06E^{-05}$
TRINITY_DN132755_c0_g1	Uncharacterized protein (*Lottia gigantea*)	V4BE89_LOTGI	$1.90E^{-12}$
TRINITY_DN209435_c0_g1			$5.13E^{-04}$
TRINITY_DN36379_c0_g1			$2.46E^{-04}$

TRINITY_DN154992_c0_g1	Uncharacterized protein (*Lottia gigantea*)	V4BE89_LOTGI	$2.78E^{-05}$
TRINITY_DN177384_c6_g1	Delta-like protein 1 (*Rattus norvegicus*)	DLL1_RAT	$2.64E^{-08}$
TRINITY_DN165965_c0_g1			$1.17E^{-07}$
TRINITY_DN162613_c0_g4			$1.10E^{-04}$
TRINITY_DN171494_c0_g2			$2.41E^{-04}$
TRINITY_DN153661_c0_g1	Uncharacterized protein (*Lottia gigantea*)	V4AXA0_LOTGI	$7.40E^{-06}$
TRINITY_DN181007_c7_g4			$2.09E^{-04}$
TRINITY_DN181264_c6_g1	Uncharacterized protein (*Arion vulgaris*)	A0A0B7ARG1_9EUPU	$1.71E^{-08}$
TRINITY_DN174156_c1_g2	Carbonic anhydrase 12 (*Mus musculus*)	CAH12_MOUSE	$3.92E^{-05}$
TRINITY_DN126771_c0_g1	Uncharacterized protein (*Crassostrea gigas*)	K1QCL4_CRAGI	$2.09E^{-04}$
TRINITY_DN154918_c0_g1			$4.57E^{-06}$
TRINITY_DN171240_c0_g1	Protein glutamine gamma glutamyltransferase K (TG) (*Canis lupus familiaris*)	TGM1_CANLF	$6.00E^{-04}$
TRINITY_DN161552_c1_g5	Follistatin A (*Danio rerio*)	FSTA_DANRE	$4.84E^{-04}$
TRINITY_DN181242_c1_g3			$2.77E^{-04}$
TRINITY_DN164779_c2_g2	RS-rich protein 1(*Mytilus coruscus*)	A0A0K0YAY1_MYTCO	$1.65E^{-06}$

TRINITY_DN136857_c0_g1	Stress induced protein 1(*Caenorhabditis elegans*)	SIP1_CAEEL	$3.25E^{-04}$
TRINITY_DN165794_c0_g5			$2.01E^{-05}$
TRINITY_DN147120_c0_g4			$2.80E^{-05}$
TRINITY_DN136535_c0_g1	Uncharacterized protein (*Lottia gigantea*)	V4BE89_LOTGI	$3.08E^{-08}$
TRINITY_DN173208_c0_g1			$1.40E^{-06}$
TRINITY_DN174212_c0_g2	Filamin A (*Crassostrea gigas*)	K1RZ99_CRAGI	$3.81E^{-06}$
TRINITY_DN172256_c0_g2			$3.79E^{-04}$
TRINITY_DN168269_c1_g4			$2.99E^{-06}$
TRINITY_DN39776_c1_g1	Tubulin β chain (*Lytechinus pictus*)	TBB_LYTPI	$1.36E^{-04}$
TRINITY_DN178147_c0_g1	Sushi von Willebrand factor type A EGF and pentraxin domain containing protein 1 (*Rattus norvegicus*)	SVEP1_RAT	$8.85E^{-09}$
TRINITY_DN163507_c2_g5	Cathepsin Z (*Crassostrea gigas*)	K1P5K4_CRAGI	$1.68E^{-05}$
TRINITY_DN55733_c0_g1			$9.30E^{-10}$
TRINITY_DN180672_c0_g6			$1.00E^{-04}$
TRINITY_DN160057_c0_g2	3-oxoacyl reductase FabG (*Thermotoga maritima*)	FABG_THEMA	$5.39E^{-08}$
TRINITY_DN130432_c0_g1			$4.59E^{-04}$
TRINITY_DN172326_c0_g3	P3 protein (*Mus musculus*)	P3_MOUSE	$3.06E^{-04}$
TRINITY_DN168269_c1_g5			$1.36E^{-04}$

TRINITY_DN119839_c0_g1	Transforming growth factor β induced protein ig-H3 (*Crassostrea gigas*)	K1PXW8_CRAGI	$2.90E^{-04}$
TRINITY_DN166617_c0_g1			$7.70E^{-05}$
TRINITY_DN180979_c1_g2			$4.71E^{-07}$
TRINITY_DN169712_c1_g3	Transgelin like protein 6 (*Mytilus galloprovincialis*)	A0A0K0YB42_MYTGA	$9.55E^{-06}$
TRINITY_DN178271_c1_g1	Calbindin 32 (*Drosophila melanogaster*)	CAB32_DROME	$8.09E^{-04}$
TRINITY_DN163693_c0_g3			$9.44E^{-06}$
TRINITY_DN124911_c0_g1			$4.57E^{-06}$
TRINITY_DN177904_c5_g1	Proline serine threonine phosphatase interacting protein 1 (*Mus musculus*)	PPIP1_MOUSE	$2.77E^{-04}$
TRINITY_DN156801_c0_g1	Protein Wnt-9a (*Gallus gallus*)	WNT9A_CHICK	$9.31E^{-04}$
TRINITY_DN151831_c0_g1	Uncharacterized protein (*Lottia gigantea*)	V4ADI7_LOTGI	$1.06E^{-08}$
TRINITY_DN165101_c2_g2	Excitatory amino acid transporter 2 (*Mus musculus*)	EAA2_MOUSE	$6.70E^{-05}$
TRINITY_DN170242_c0_g1	Uncharacterized protein (*Lottia gigantea*)	V4AFU8_LOTGI	$1.07E^{-04}$
TRINITY_DN111179_c0_g1	Insulin gene enhancer protein ISL-3 (*Oncorhynchus tshawytscha*)	ISL3_ONCTS	$2.52E^{-04}$

TRINITY_DN165794_c0_g8	Adipocyte plasma membrane-associated protein (*Bos taurus*)	APMAP_BOVIN	$5.41E^{-09}$
TRINITY_DN181243_c4_g2			$1.12E^{-05}$
TRINITY_DN181242_c2_g3			$9.44E^{-06}$
TRINITY_DN151365_c0_g1			$4.30E^{-04}$
TRINITY_DN80739_c0_g1			$7.65E^{-05}$
TRINITY_DN165520_c0_g2	Solute carrier organic anion transporter family member 4A1 (*Mus musculus*)	SO4A1_MOUSE	$3.82E^{-08}$
TRINITY_DN171085_c0_g2	Insulinoma associated protein 1a (*Danio rerio*)	INS1A_DANRE	$3.68E^{-05}$
TRINITY_DN180241_c1_g8	Myosin heavy chain striated muscle (*Argopecten irradians*)	MYS_ARGIR	$6.20E^{-08}$
TRINITY_DN168287_c2_g1			$4.18E^{-04}$
TRINITY_DN172809_c0_g3	Uncharacterized protein (*Crassostrea gigas*)	K1RAV4_CRAGI	$1.79E^{-04}$
TRINITY_DN160389_c0_g2			$9.99E^{-05}$
TRINITY_DN160241_c3_g4	Retinal-binding protein (*Todarodes pacificus*)	RALB_TODPA	$1.72E^{-04}$
TRINITY_DN169536_c0_g2	Asparagine synthetase (*Gallus gallus*)	ASNS_CHICK	$1.57E^{-04}$
TRINITY_DN164018_c0_g6	Telokin (*Meleagris gallopavo*)	MYLK_MELGA	$2.26E^{-04}$
TRINITY_DN167023_c0_g1	Uncharacterized protein (*Crassostrea gigas*)	K1QA71_CRAGI	$1.43E^{-09}$

TRINITY_DN157911_c0_g7			$2.27E^{-05}$
TRINITY_DN163774_c0_g1	Uncharacterized protein (*Crassostrea gigas*)	K1RCI7_CRAGI	$3.15E^{-04}$
TRINITY_DN170121_c0_g1	Deleted in malignant brain tumors 1 protein (*Crassostrea gigas*)	K1QKF1_CRAGI	$5.00E^{-04}$
TRINITY_DN170676_c0_g4	α-mannosidase 2 (*Homo sapiens*)	MA2A1_HUMAN	$1.14E^{-05}$
TRINITY_DN147565_c0_g2			$6.65E^{-04}$
TRINITY_DN181007_c6_g2			$5.24E^{-06}$
TRINITY_DN113020_c0_g1			$1.09E^{-05}$
TRINITY_DN175418_c0_g4	Atrial natriuretic peptide converting enzyme (*Homo sapiens*)	CORIN_HUMAN	$1.20E^{-05}$
TRINITY_DN168060_c0_g1	Uncharacterized protein (*Crassostrea gigas*)	K1REI1_CRAGI	$1.68E^{-06}$
TRINITY_DN155034_c0_g2	Cysteine and glycine-rich protein 2 (*Coturnix coturnix japonica*)	CSRP2_COTJA	$1.68E^{-05}$
TRINITY_DN180601_c6_g4	Prestin (*Homo sapiens*)	S26A5_HUMAN	$3.61E^{-08}$
TRINITY_DN177208_c0_g1	Protein FAM46C (*Danio rerio*)	FA46C_DANRE	$5.75E^{-05}$
TRINITY_DN169640_c1_g6			$6.96E^{-08}$
TRINITY_DN181242_c2_g2	Twitchin (*Mytilus galloprovincialis*)	Q9GV22_MYTGA	$8.09E^{-07}$
TRINITY_DN172176_c0_g2	Neuronal calcium sensor 1 (*Aplysia californica*)	NCS1_APLCA	$5.17E^{-05}$

TRINITY_DN180542_c2_g2			$6.34E^{-06}$
TRINITY_DN180672_c0_g3			$3.43E^{-05}$
TRINITY_DN167040_c2_g1	Putative methyltransferase NSUN7 (*Homo sapiens*)	NSUN7_HUMAN	$8.03E^{-04}$
TRINITY_DN163304_c0_g1	Microtubule-associated protein (*Lottia gigantea*)	V4A5P5_LOTGI	$2.64E^{-08}$
TRINITY_DN162527_c0_g1			$1.10E^{-06}$
TRINITY_DN178644_c0_g1	Adenylate cyclase type 1 (Ca/calmodulin-activated adenylyl cyclase (*Mus musculus*)	ADCY1_MOUSE	$1.27E^{-08}$
TRINITY_DN143151_c0_g1	Ceroid-lipofuscinosis neuronal protein 5 homolog (*Mus musculus*)	CLN5_MOUSE	$1.18E^{-04}$
TRINITY_DN178495_c2_g1			$3.59E^{-06}$
TRINITY_DN181242_c3_g1	Twitchin (*Mytilus galloprovincialis*)	Q7YT99_MYTGA	$1.02E^{-05}$
TRINITY_DN181009_c3_g1	Inositol 1,4,5-trisphosphate receptor type 1 (*Mus musculus*)	ITPR1_MOUSE	$8.76E^{-04}$
TRINITY_DN160241_c2_g1	Retinal-binding protein (*Todarodes pacificus*)	RALB_TODPA	$6.70E^{-05}$
TRINITY_DN169132_c1_g3	Solute carrier family 15 member 4 (*Xenopus laevis*)	S15A4_XENLA	$7.86E^{-05}$
TRINITY_DN155034_c0_g3			$5.52E^{-04}$
TRINITY_DN173511_c0_g5	Von Willebrand factor D and EGF domain-	K1P6L3_CRAGI	$6.29E^{-04}$

	containing protein (*Crassostrea gigas*)		
TRINITY_DN165888_c0_g2	trembl- - Chitin_deacetylase-like_protein-1_(*Mytilus_coruscus_*)	A0A0K0YAZ8_MYTCO	$1.02E^{-05}$
TRINITY_DN164744_c0_g1	-sprot- - Paramyosin(*Mytilus_galloprovincialis*)	MYSP_MYTGA	$8.42E^{-07}$
TRINITY_DN145016_c0_g1			$6.46E^{-05}$
TRINITY_DN159230_c0_g2	Uncharacterized protein (*Arion vulgaris*)	A0A0B6ZGK1_9EUPU	$5.17E^{-05}$
TRINITY_DN164980_c0_g1	Potassium voltage-gated channel protein Shaw (*Drosophila melanogaster*)	KCNAW_DROME	$5.49E^{-04}$
TRINITY_DN165814_c1_g3			$4.94E^{-04}$
TRINITY_DN158951_c0_g1	Leucine-rich repeat-containing protein 74A (*Homo sapiens*)	LR74A_HUMAN	$1.79E^{-04}$
TRINITY_DN171746_c0_g5	Major egg antigen (*Crassostrea gigas*)	K1QBC3_CRAGI	$2.30E^{-05}$
TRINITY_DN165306_c0_g1	Kyphoscoliosis peptidase (*Crassostrea gigas*)	K1PQ64_CRAGI	$5.74E^{-07}$
TRINITY_DN173216_c0_g1	Voltage-gated hydrogen channel 1 (*Gallus gallus*)	HVCN1_CHICK	$1.92E^{-05}$
TRINITY_DN152699_c0_g1			$7.58E^{-04}$
TRINITY_DN180224_c1_g1	Uncharacterized protein (*Crassostrea gigas*)	K1PYE4_CRAGI	$6.16E^{-09}$

TRINITY_DN169711_c0_g1	Neurotrypsin (*Crassostrea gigas*)	K1R3V1_CRAGI	$9.44E^{-06}$
TRINITY_DN168946_c1_g5	Transgelin-like protein-3(*Mytilus coruscus*)	A0A0K0YAZ6_MYTCO	$9.46E^{-04}$
TRINITY_DN169640_c1_g4			$5.00E^{-04}$
TRINITY_DN176716_c1_g1	Whirlin (*Crassostrea gigas*)	K1QHV6_CRAGI	$5.79E^{-06}$
TRINITY_DN160107_c0_g2	Uncharacterized protein (*Crassostrea gigas*)	K1Q4Q0_CRAGI	$1.31E^{-04}$
TRINITY_DN171546_c0_g3	Matrix metalloproteinase-19 (*Homo sapiens*)	MMP19_HUMAN	$1.17E^{-06}$
TRINITY_DN156972_c0_g2	Aquaporin-4 (*Mus musculus*)	AQP4_MOUSE	$6.58E^{-07}$
TRINITY_DN108822_c0_g1	Uncharacterized protein (*Crassostrea gigas*)	K1Q679_CRAGI	$1.08E^{-04}$
TRINITY_DN165433_c1_g11			$9.40E^{-04}$
TRINITY_DN141471_c0_g1	Forkhead box protein F1 (*Crassostrea gigas*)	K1QD97_CRAGI	$2.67E^{-06}$
TRINITY_DN158052_c0_g3	Uncharacterized protein (*Lottia gigantea*)	V4B5G7_LOTGI	$7.10E^{-04}$
TRINITY_DN169640_c1_g5			$6.01E^{-04}$
TRINITY_DN153182_c0_g11	Four and a half LIM domains protein 2 (*Rattus norvegicus*)	FHL2_RAT	$1.28E^{-05}$
TRINITY_DN178500_c2_g1	Uncharacterized protein (*Lottia gigantea*)	V4B719_LOTGI	$2.31E^{-04}$

TRINITY_DN173065_c1_g8	Receptor-transporting protein 3 (*Crassostrea gigas*)	K1Q6J6_CRAGI	$7.11E^{-04}$
TRINITY_DN141305_c0_g1	CTL-9 (*Argopecten irradians*)	H9M3L7_ARGIR	$9.06E^{-04}$
TRINITY_DN159435_c1_g3	NACHT and WD repeat domain containing protein 1 (*Crassostrea gigas*)	K1PEH6_CRAGI	$1.31E^{-06}$
TRINITY_DN147647_c0_g1	Cysteine-rich protein 1 (*Homo sapiens*)	CRIP1_HUMAN	$8.32E^{-05}$
TRINITY_DN171746_c0_g8	Major egg antigen (*Schistosoma mansoni*)	P40_SCHMA	$5.66E^{-04}$
TRINITY_DN171622_c1_g5			$4.70E^{-05}$
TRINITY_DN150421_c0_g3	Endoprotease bli-4 (*Caenorhabditis elegans*)	BLI4_CAEEL	$3.65E^{-04}$
TRINITY_DN169884_c0_g1			$6.10E^{-05}$
TRINITY_DN180343_c1_g3			$1.72E^{-04}$
TRINITY_DN178827_c2_g3	Uncharacterized protein (*Lottia gigantea*)	V4A8I7_LOTGI	$1.91E^{-04}$
TRINITY_DN165868_c1_g2	Lipopolysaccharide-induced tumor necrosis factor-α factor homolog (*Gallus gallus*)	LITAF_CHICK	$8.38E^{-04}$
TRINITY_DN151425_c0_g3			$1.45E^{-05}$
TRINITY_DN180618_c0_g1	Filamin-A (*Homo sapiens*)	FLNA_HUMAN	$1.57E^{-05}$
TRINITY_DN158735_c0_g1	Xaa-Pro aminopeptidase 1 (*Homo sapiens*)	XPP1_HUMAN	$5.13E^{-04}$

TRINITY_DN149941_c0_g1	Uncharacterized protein (*Lottia gigantea*)	V3ZVL6_LOTGI	$1.41E^{-04}$
TRINITY_DN163285_c0_g1			$3.77E^{-04}$
TRINITY_DN175808_c0_g1			$3.19E^{-04}$
TRINITY_DN175789_c0_g1	Multidrug resistance-associated protein 1 (Leukotriene C transporter) (*Canis lupus familiaris*)	MRP1_CANLF	$1.36E^{-04}$
TRINITY_DN181321_c5_g1			$8.68E^{-04}$
TRINITY_DN143314_c0_g2			$8.03E^{-04}$
TRINITY_DN155835_c0_g1	Sarcoplasmic calcium-binding protein (*Mizuhopecten yessoensis*)	SCP_MIZYE	$1.00E^{-07}$
TRINITY_DN171569_c1_g1	Tripartite motif-containing protein 56 (*Crassostrea gigas*)	K1QSK1_CRAGI	$6.20E^{-06}$
TRINITY_DN70925_c0_g1	Uncharacterized protein (*Crassostrea gigas*)	K1PAP3_CRAGI	$6.70E^{-05}$
TRINITY_DN174264_c0_g1	Somatomedin-B and thrombospondin type-1 domain-containing protein (*Bos taurus*)	SBSPO_BOVIN	$2.20E^{-07}$
TRINITY_DN179350_c2_g4	PDZ and LIM domain protein 5(*Crassostrea gigas*)	K1PQ23_CRAGI	$1.59E^{-04}$
TRINITY_DN175603_c0_g1	Receptor-type tyrosine-protein phosphatase ε (*Homo sapiens*)	PTPRE_HUMAN	$1.84E^{-04}$
TRINITY_DN181141_c7_g1	Von Willebrand factor D and EG domain-	K1RSH8_CRAGI	$6.75E^{-07}$

	containing protein (*Crassostrea gigas*)		
TRINITY_DN180790_c4_g3	Muscle M-line assembly protein unc-89 (*Crassostrea gigas*)	K1RUG0_CRAGI	$3.58E^{-04}$
TRINITY_DN158068_c0_g3	Actin-2 (*Lumbricus terrestris*)	ACT2_LUMTE	$6.84E^{-06}$
TRINITY_DN40608_c0_g2			$9.17E^{-04}$
TRINITY_DN178763_c1_g1			$1.90E^{-04}$
TRINITY_DN176510_c1_g2	Kyphoscoliosis peptidase (*Mus musculus*)	KY_MOUSE	$1.57E^{-05}$
TRINITY_DN137215_c0_g1	Uridine phosphorylase 1 (*Homo sapiens*)	UPP1_HUMAN	$2.09E^{-04}$
TRINITY_DN176604_c0_g2	Membrane metallo-endopeptidase-like 1 (NEP2) (*Homo sapiens*)	MMEL1_HUMAN	$6.69E^{-06}$
TRINITY_DN179325_c0_g2	Fibulin-2 (*Mus musculus*)	FBLN2_MOUSE	$4.01E^{-04}$
TRINITY_DN165677_c4_g1			$6.44E^{-04}$
TRINITY_DN181271_c0_g2	Dystonin (*Mus musculus*)	DYST_MOUSE	$3.09E^{-06}$
TRINITY_DN157598_c0_g1	Junctophilin-1 (*Mus musculus*)	JPH1_MOUSE	$7.40E^{-06}$
TRINITY_DN171593_c0_g1	Protein fem-1 homolog C (*Danio rerio*)	FEM1C_DANRE	$4.89E^{-04}$
TRINITY_DN159603_c0_g2	Inhibitory POU protein (*Drosophila melanogaster*)	IPOU_DROME	$3.89E^{-04}$

TRINITY_DN157616_c1_g5	Myophilin (*Echinococcus granulosus*)	MYPH_ECHGR	$4.94E^{-04}$
TRINITY_DN180244_c4_g2	Uncharacterized protein (*Crassostrea gigas*)	K1Q3F5_CRAGI	$6.00E^{-04}$
TRINITY_DN167783_c1_g6	Actin adductor muscle (*Placopecten magellanicus*)	ACT_PLAMG	$4.00E^{-05}$
TRINITY_DN174226_c0_g5	Sodium and chloride-dependent glycine transporter 2 (*Homo sapiens*)	SC6A5_HUMAN	$1.12E^{-05}$
TRINITY_DN180737_c4_g1	Polypeptide N-acetylgalactosaminyltransferase 1 (*Homo sapiens*)	GALT1_HUMAN	$1.67E^{-05}$
TRINITY_DN174419_c2_g5	Aquaporin-4 (*Bos taurus*)	AQP4_BOVIN	$3.09E^{-06}$
TRINITY_DN172100_c0_g1	Synaptopodin-2 (*Crassostrea gigas*)	K1Q2X5_CRAGI	$1.71E^{-08}$
TRINITY_DN128736_c0_g1	Peroxidasin-like protein (*Crassostrea gigas*)	K1PG33_CRAGI	$9.34E^{-04}$
TRINITY_DN176703_c1_g2	Voltage-dependent L-type calcium channel subunit α (*Crassostrea gigas*)	K1QEL8_CRAGI	$3.08E^{-08}$
TRINITY_DN170085_c0_g1	Glutamate receptor ionotropic NMDA 2B (*Homo sapiens*)	NMDE2_HUMAN	$7.25E^{-04}$
TRINITY_DN173994_c1_g10	Troponin I (*Chlamys nipponensis akazara*)	TNNI_CHLNI	$5.13E^{-04}$
TRINITY_DN175773_c0_g2	Potassium voltage-gated channel protein	KCNAL_DROME	$5.17E^{-05}$

	Shal (*Drosophila melanogaster*)		
TRINITY_DN170789_c0_g1	Uncharacterized protein (*Crassostrea gigas*)	K1Q856_CRAGI	$1.40E^{-04}$
TRINITY_DN175685_c1_g2	Uncharacterized protein (*Arion vulgaris*)	A0A0B6YXZ9_9EUPU	$2.09E^{-04}$
TRINITY_DN164856_c3_g1	Calponin-1 (*Gallus gallus*)	CNN1_CHICK	$8.22E^{-04}$
TRINITY_DN169884_c0_g3			$1.14E^{-04}$
TRINITY_DN151756_c0_g1	Integrin β-5 (*Bos taurus*)	ITB5_BOVIN	$1.86E^{-04}$
TRINITY_DN134224_c0_g1	Insulin gene enhancer protein ISL-1 (*Rattus norvegicus*)	ISL1_RAT	$5.66E^{-04}$
TRINITY_DN162423_c0_g3	Small conductance calcium-activated potassium channel protein 2 (*Homo sapiens*)	KCNN2_HUMAN	$8.93E^{-04}$
TRINITY_DN160540_c3_g2	Sodium-coupled monocarboxylate transporter 1 (*Homo sapiens*)	SC5A8_HUMAN	$7.31E^{-04}$
TRINITY_DN179779_c5_g2			$4.61E^{-07}$
TRINITY_DN181260_c4_g1	LIM domain and actin-binding protein 1 (*Crassostrea gigas*)	K1QT91_CRAGI	$3.79E^{-04}$
TRINITY_DN179098_c3_g3	Uncharacterized protein (*Crassostrea gigas*)	K1RE15_CRAGI	$8.03E^{-04}$
TRINITY_DN137669_c0_g1			$2.78E^{-04}$

TRINITY_DN180712_c8_g2			$1.01E^{-05}$
TRINITY_DN170528_c0_g1	Inositol-3-phosphate synthase 1-B (*Xenopus laevis*)	INO1B_XENLA	$5.63E^{-06}$
TRINITY_DN147265_c0_g1	Heart and neural crest derivatives expressed protein 2 (*Danio rerio*)	HAND2_DANRE	$2.90E^{-04}$
TRINITY_DN63689_c0_g1			$4.12E^{-05}$
TRINITY_DN179234_c0_g4	Trafficking protein particle complex subunit 4 (*Crassostrea gigas*)	K1QNE7_CRAGI	$1.91E^{-04}$
TRINITY_DN179091_c4_g1			$2.20E^{-05}$
TRINITY_DN144067_c0_g2			$2.78E^{-04}$
TRINITY_DN153348_c0_g1			$1.07E^{-04}$
TRINITY_DN159919_c2_g3			$5.30E^{-04}$
TRINITY_DN151657_c0_g1	Uncharacterized protein (*Crassostrea gigas*)	K1RQH6_CRAGI	$5.37E^{-04}$
TRINITY_DN169166_c0_g1	WEE1-like protein kinase 1-B (*Xenopus laevis*)	WEE1B_XENLA	$2.20E^{-06}$
TRINITY_DN179730_c7_g3			$1.23E^{-04}$
TRINITY_DN109592_c0_g1	Collagen α-1 chain (*Crassostrea gigas*)	K1R0L2_CRAGI	$8.16E^{-05}$
TRINITY_DN179876_c1_g8			$6.35E^{-04}$
TRINITY_DN151440_c0_g4			$2.62E^{-09}$
TRINITY_DN167317_c0_g3	Prestin (*Mus musculus*)	S26A5_MOUSE	$6.16E^{-09}$

Supplementary Table S6. Expression profiles of contigs encoding SMPs in Baltic *M. edulis*-like larvae. EE refers to equally expressed.

SMPs and SMP related domains	Contig_ID	Expression profile	Maximum posterior probability
α_CA_(Nacrein)	TRINITY_DN165584_c2_g1	Down-Up-Up-Up-Up	0.5072
α _CA_(Nacrein)	TRINITY_DN170492_c0_g2	Up-Down-Down-Down-Down	1
α _CA_(Nacrein)	TRINITY_DN172452_c1_g4	EE-EE-Up-Up-EE	0.4648
α _CA_(Nacrein)	TRINITY_DN174156_c1_g2	Down-Up-Up-Up-Down	0.4826
Amine_oxidase	TRINITY_DN181138_c7_g5	Down-Up-Up-Up-Up	0.872
β _Thymosin	TRINITY_DN170261_c1_g2	Down-Up-Up-Up-Up	0.9223
B-hexosaminodase	TRINITY_DN158110_c0_g2	Down-Up-Up-Up-Up	0.7494
B-hexosaminodase	TRINITY_DN161171_c0_g1	Down-Up-Up-Up-Up	0.562
B-hexosaminodase	TRINITY_DN165446_c0_g1	Down-Up-Down-Down-Down	0.4996
B-hexosaminodase	TRINITY_DN177006_c2_g4	Down-Up-Up-Up-Up	0.6592
B-hexosaminodase	TRINITY_DN177547_c1_g1	Down-Up-Up-Up-Up	0.8364
B-lactamase_(Gigasin6)	TRINITY_DN175515_c2_g6	Up-Down-Down-Down-Down	0.7507
B-lactamase_(Gigasin6)	TRINITY_DN176457_c3_g4	Down-Down-Up-Up-Down	0.4305
BPTI_Kunitz	TRINITY_DN151020_c0_g1	Down-Up-Up-Up-Down	0.913
BPTI_Kunitz	TRINITY_DN161426_c0_g1	Down-Up-Up-Up-Up	0.8287
BPTI_Kunitz	TRINITY_DN168983_c0_g2	Up-Down-Down-Down-Down	0.5696
BPTI_Kunitz	TRINITY_DN178763_c1_g1	Down-Up-Up-Up-Down	0.5
BPTI_Kunitz	TRINITY_DN178763_c2_g3	Down-Up-Up-Down-Down	0.5
BPTI_Kunitz	TRINITY_DN178763_c2_g4	Down-Up-Up-Down-Down	0.5
BPTI_Kunitz	TRINITY_DN180147_c1_g1	Up-Down-Up-Up-Down	0.4711
BPTI_Kunitz	TRINITY_DN59474_c0_g1	Down-Up-Up-Down-Up	0.3917

BPTI_Kunitz	TRINITY_DN73045_c0_g2	Down-Up-Up-Up-Down	0.4874
C1Q	TRINITY_DN155170_c0_g2	EE-EE-Down-Down-Down	0.3437
C1Q	TRINITY_DN81311_c0_g1	Down-Up-Up-Up-Up	0.9457
Chitin_binding_(Sushi)	TRINITY_DN178147_c0_g1	Down-Up-Up-Up-Down	0.4996
Chitin_binding_(VWA)	TRINITY_DN177076_c0_g1	Down-Up-Up-Up-Down	0.8702
Chitin_Synthase_(Myosin-Ploop)	TRINITY_DN180880_c4_g1	Down-Up-Down-Up-Up	0.4999
C-lectin_(Perlucin)	TRINITY_DN107937_c0_g1	Down-Up-Up-Up-Up	0.1899
C-lectin_(Perlucin)	TRINITY_DN111847_c0_g1	Down-Up-Up-Up-Up	0.9678
C-lectin_(Perlucin)	TRINITY_DN117910_c0_g1	Down-Up-Up-Up-Up	0.8755
C-lectin_(Perlucin)	TRINITY_DN141305_c0_g1	Down-Up-Up-Up-Up	0.9746
C-lectin_(Perlucin)	TRINITY_DN146282_c1_g1	EE-EE-Up-Down-EE	0.1955
C-lectin_(Perlucin)	TRINITY_DN156726_c0_g2	Down-Up-Up-Up-Up	0.504
C-lectin_(Perlucin)	TRINITY_DN160524_c5_g2	Down-Down-Down-Down-Down	0.2449
C-lectin_(Perlucin)	TRINITY_DN173794_c0_g2	Down-Up-Up-Up-Up	0.4792
C-lectin_(Perlucin)	TRINITY_DN179775_c0_g2	EE-Down-Down-Down-Down	0.4614
C-lectin_(Perlucin)	TRINITY_DN180930_c1_g1	Down-Up-Up-Up-Up	0.3908
Concanavalin_A	TRINITY_DN177325_c0_g1	Down-Up-Down-Down-Down	0.8594
Cyclophilin_PPIase	TRINITY_DN154373_c0_g2	Down-Down-Down-Down-Down	0.5
Cyclophilin_PPIase	TRINITY_DN157791_c0_g1	Up-Down-Down-Up-Down	0.4982
Cyclophilin_PPIase	TRINITY_DN162724_c0_g1	Up-Down-Down-Up-Down	0.4998
Cyclophilin_PPIase	TRINITY_DN167409_c0_g4	Down-EE-EE-EE-Down	0.4087
Cyclophilin_PPIase	TRINITY_DN169298_c1_g1	Up-Down-Down-Up-Down	0.3218
Cyclophilin_PPIase	TRINITY_DN171027_c1_g12	Down-Up-Up-Up-Down	0.5

Dermatopontin	TRINITY_DN163610_c2_g3	Down-Down-Down-Down-Down	0.9441
Dopamine_B-h-ase	TRINITY_DN147142_c0_g1	Down-Up-Up-Up-Up	0.5297
Dopamine_B-h-ase	TRINITY_DN167284_c0_g1	Down-Up-Up-Up-Up	0.9664
Dopamine_B-h-ase	TRINITY_DN172693_c0_g5	Up-Down-Down-Down-Down	0.4659
Dopamine_B-h-ase	TRINITY_DN175579_c4_g2	Up-Down-Down-Down-Down	0.4912
Fibronectin_III_(IG)	TRINITY_DN170889_c0_g5	Down-Up-Up-Up-Up	0.4638
Fibronectin_III_(IG)	TRINITY_DN171964_c0_g1	Down-Up-Up-Down-Down	0.5
Fibronectin_III_(IG)	TRINITY_DN174919_c0_g6	Down-Down-Up-Up-Down	0.4983
Fibronectin_III_(IG)	TRINITY_DN174919_c0_g7	Down-Down-Up-Up-Down	0.4949
Fibronectin_III_(IG)	TRINITY_DN180108_c1_g1	Up-Down-Down-Down-Up	0.4989
Fibronectin_III_(IG)	TRINITY_DN180472_c4_g4	Up-Down-Up-Up-Up	0.4117
Fibronectin_III_(IG)	TRINITY_DN180652_c2_g2	Down-Up-Up-Up-Up	0.4998
Fibronectin_III_(IG)	TRINITY_DN180733_c3_g2	Down-Up-EE-EE-EE	0.4998
Fibronectin_III_(IG)	TRINITY_DN181145_c5_g1	Down-Down-Down-Down-Up	0.4939
Fibronectin_III_(IG)	TRINITY_DN181243_c4_g2	Down-Up-Up-Up-Down	0.6769
Filament_(Filamin)	TRINITY_DN158160_c0_g2	Down-Up-Up-Down-Down	0.5018
Filament_(Filamin)	TRINITY_DN170104_c0_g5	Down-Up-Up-Up-Down	0.5
Filament_(Filamin)	TRINITY_DN174754_c0_g1	EE-Down-EE-EE-EE	0.499
Filament_(Filamin)	TRINITY_DN174767_c1_g7	Down-Up-Up-Down-Down	0.5
Filament_(Filamin)	TRINITY_DN177697_c2_g2	Down-Up-Up-Down-Down	0.5008
Filament_(Filamin)	TRINITY_DN178513_c0_g1	Up-Down-Down-Down-Up	0.4949
Filament_(Filamin)	TRINITY_DN180618_c0_g1	Down-Up-Up-Down-Down	0.5
Filament_(Filamin)	TRINITY_DN180761_c0_g3	Down-Up-Down-Down-Down	0.5
Filament_(Filamin)	TRINITY_DN181051_c4_g1	Down-Up-Up-Up-Up	0.5

Filament_(Filamin)	TRINITY_DN181268_c3_g2	Down-Up-Up-Down-Down	0.5
Filament_(Filamin)	TRINITY_DN181271_c0_g2	Down-Up-Up-Down-Up	0.5
Filament_(Filamin)	TRINITY_DN181327_c8_g1	Down-Down-Up-Up-Up	0.4961
Filament_(Filamin)	TRINITY_DN181331_c4_g1	Down-Up-Up-Down-Up	0.5
Gigasin_3a	TRINITY_DN134486_c0_g1	Down-Up-Up-Up-Up	0.6087
Gly-Hyd_(Chitinase)	TRINITY_DN156856_c2_g1	Down-Up-Up-Up-Down	0.7134
Gly-Hyd_(Chitinase)	TRINITY_DN162472_c0_g3	Down-Up-Up-Up-Up	0.4053
Gly-Hyd_(Chitinase)	TRINITY_DN162472_c0_g4	Down-Up-Up-Up-Up	0.5383
Gly-Hyd_(Chitinase)	TRINITY_DN165152_c0_g1	Down-Up-Up-Up-Up	0.5362
Gly-Hyd_(Chitinase)	TRINITY_DN166699_c0_g2	Down-Up-Up-Up-Up	0.5666
Gly-Hyd_(Chitinase)	TRINITY_DN166785_c1_g4	Down-Up-Up-Down-Down	0.5
Gly-Hyd_(Chitinase)	TRINITY_DN167601_c0_g1	Down-Up-Up-Up-Down	0.4996
Gly-Hyd_(Chitinase)	TRINITY_DN173599_c0_g4	Up-Up-Up-Up-Up	0.3727
Gly-Hyd_(Chitinase)	TRINITY_DN173976_c1_g4	Up-Down-Down-Down-Down	0.5178
Gly-Hyd_(Chitinase)	TRINITY_DN174242_c0_g2	Down-Up-EE-EE-Down	0.4998
Haem_Peroxidase	TRINITY_DN149477_c0_g1	Down-Down-Down-Down-Down	0.7277
Haem_Peroxidase	TRINITY_DN150505_c0_g1	Down-Up-Up-Up-Up	0.4969
Haem_Peroxidase	TRINITY_DN163196_c0_g1	Down-Up-Up-Down-Down	0.4957
Haem_Peroxidase	TRINITY_DN164523_c1_g1	Down-Down-Down-Down-Down	0.5004
Haem_Peroxidase	TRINITY_DN165764_c0_g5	Down-Down-Down-Down-Down	0.422
Haem_Peroxidase	TRINITY_DN166945_c3_g1	Up-Down-Down-Down-Up	0.4718
Haem_Peroxidase	TRINITY_DN175179_c1_g2	EE-EE-EE-Down-Down	0.4827
Haem_Peroxidase	TRINITY_DN176052_c1_g6	Down-Up-Up-Down-Down	0.4957

Kazal_domain	TRINITY_DN150779_c0_g1	Up-Down-Up-Up-Up	0.4826
Kazal_domain	TRINITY_DN174904_c1_g16	Up-Down-Up-Up-Up	0.2418
Peptidase_C1A	TRINITY_DN144206_c0_g1	Down-Up-Up-Up-Up	0.6174
Peptidase_C1A	TRINITY_DN146921_c0_g1	Down-Up-Up-Up-Up	0.9842
Peptidase_C1A	TRINITY_DN162930_c0_g1	Down-Up-Up-Up-Up	0.8632
Peptidase_C1A	TRINITY_DN168131_c0_g1	Down-Up-Up-Up-Up	0.6678
Peptidase_C1A	TRINITY_DN175117_c1_g5	EE-EE-EE-Up-Up	0.4935
Peroxiredoxin	TRINITY_DN164166_c0_g2	Down-Up-Up-Up-Up	0.5032
RLCD	TRINITY_DN155318_c2_g1	Down-Up-Up-Up-Up	0.5064
RLCD	TRINITY_DN156777_c0_g1	Down-Up-Up-Up-Up	0.5252
RLCD	TRINITY_DN164779_c2_g2	Down-Up-Up-Up-Up	0.6154
RLCD	TRINITY_DN165306_c0_g1	Down-Up-Up-Up-Down	0.7192
RLCD	TRINITY_DN171531_c0_g2	Down-Up-Down-Down-Down	0.5
RLCD	TRINITY_DN176059_c0_g1	EE-EE-Up-EE-Down	0.4817
RLCD	TRINITY_DN178042_c3_g1	EE-EE-Up-Up-Up	0.4263
RLCD	TRINITY_DN178597_c2_g3	Down-Down-Down-Down-Down	0.4888
SCP-domain	TRINITY_DN147565_c0_g2	Down-Up-Up-Up-Down	0.5
Transgelin-Calponin	TRINITY_DN157616_c1_g5	Down-Up-Up-Up-Down	0.4998
Transgelin-Calponin	TRINITY_DN164856_c3_g1	Down-Up-Up-Up-Up	0.5
Transgelin-Calponin	TRINITY_DN165228_c0_g1	Up-Down-Down-Down-Up	0.5
Transgelin-Calponin	TRINITY_DN168946_c1_g5	Down-Up-Up-Up-Up	0.9045
Transgelin-Calponin	TRINITY_DN169712_c1_g3	Down-Up-Up-Up-Up	0.8298
Transgelin-Calponin	TRINITY_DN180079_c2_g4	Down-Up-Up-Up-Up	0.6561
Tyrosinase	TRINITY_DN155153_c0_g1	EE-Down-EE-Down-Down	0.3951

Tyrosinase	TRINITY_DN155544_c0_g1	Up-Down-Down-Down-Down	0.476
Tyrosinase	TRINITY_DN157611_c0_g2	Down-Up-Up-Up-Down	0.5
Tyrosinase	TRINITY_DN168278_c1_g2	Down-Up-Up-Up-Up	0.3746
Tyrosinase	TRINITY_DN174850_c0_g1	Up-Down-Up-Down-Down	0.1338
Tyrosinase	TRINITY_DN179853_c0_g1	Down-Up-Down-Down-Down	0.5
Tyrosinase	TRINITY_DN181289_c7_g1	Down-Up-Up-Down-Down	0.5
U	TRINITY_DN161545_c4_g3	Down-Up-Up-Down-Down	0.5

Supplementary Table S7. Expression profiles of contigs encoding ion transport proteins in Baltic *M. edulis*-like larvae depicted in figures 1-3. EE refers to equally expressed.

Figure	Contig_ID	Expression profile	Maximum posterior probability
1a	Anion exchanger- TRINITY_DN173786_c0_g2	Up-Down-Down-Down-Down	0.4986
1a	Anion exchanger- TRINITY_DN179651_c3_g2	Down-Up-Down-Down-Down	0.4361
1a	Anion transporter- TRINITY_DN165520_c0_g2	Down-Up-Up-Up-Down	0.5008
1a	Anion transporter- TRINITY_DN166908_c0_g4	Up-Down-Down-Down-Down	0.467
1a	Anion transporter- TRINITY_DN169687_c0_g2	Down-Up-Up-Down-Down	0.5
1b	Sodium bicarbonate cotransporter- TRINITY_DN175808_c1_g5	Down-Up-Down-Down-Down	0.6333
1c	Electrogenic sodium bicarbonate cotransporter- TRINITY_DN95691_c0_g1	Down-Up-Down-Down-Down	0.8781
1c	Electroneutral sodium bicarbonate exchanger- TRINITY_DN166271_c2_g1	Up-Down-Down-Down-Up	0.5047
1c	Electroneutral sodium bicarbonate exchanger- TRINITY_DN167998_c0_g2	EE-Down-Down-EE-Up	0.449
1c	Electroneutral sodium bicarbonate exchanger- TRINITY_DN176772_c2_g1	Down-Up-Up-Up-Down	0.3413
1d	Prestin- TRINITY_DN173725_c0_g1	Down-Up-Up-Up-Down	0.5291
2a	Calcium transporting ATPase- TRINITY_DN170482_c0_g1	Up-Down-Down-Down-Up	0.4454
2a	Plasma membrane calcium transporting ATPase- TRINITY_DN179905_c2_g1	Down-Down-Down-Down-Down	0.5
2a	SERCA- TRINITY_DN155835_c0_g1	Down-Up-Up-Up-Up	0.5
2a	SERCA-TRINITY_DN174921_c2_g3	Down-Up-Up-Up-Up	0.5147
2b	Sodium/Calcium exchanger- TRINITY_DN176141_c2_g2	EE-Up-Up-EE-EE-	0.4824
2b	Sodium/Calcium exchanger- TRINITY_DN171813_c0_g4	Down-Up-EE-Down-EE	0.4313
2b	Sodium/Calcium exchanger- TRINITY_DN174400_c0_g2	Up-Down-Down-Up-Up	0.4992

3a	NKA alpha subunit-TRINITY_DN173934_c0_g2	Down-Down-Down-Down-Down	0.5005
3a	NKA beta subunit-TRINITY_DN169083_c0_g2	Up-Down-Down-Down-Down	0.5126
3b	Mitochondrial NHE-TRINITY_DN165050_c0_g1	Down-Down-Down-Down-Down	0.4981
3b	Mitochondrial NHE-TRINITY_DN178761_c3_g2	Down-Down-Down-Down-Down	0.5002
3b	NHE regulatory cofactor-TRINITY_DN178396_c3_g2	Down-Up-Up-Up-Up	0.5
3c	Inward rectifier potassium channel-TRINITY_DN157228_c0_g1	Up-Down-Down-Down-Down	0.7796
3c	Inward rectifier potassium channel-TRINITY_DN159142_c1_g1	Down-Up-Up-Down-Down	0.4989
3d	Calcium activated chloride channel-TRINITY_DN166073_c0_g1	Down-Up-Up-Up-Up	0.4653
3d	Chloride channel-TRINITY_DN176195_c1_g1	Down-Up-Up-Up-Up	0.5059
3d	Intracellular chloride channel-TRINITY_DN177659_c2_g3	Down-Up-Up-Up-Up	0.5808
